# A B‐cell or a key player? The different roles of B‐cells and antibodies in melanoma

**DOI:** 10.1111/pcmr.13031

**Published:** 2022-03-04

**Authors:** Chloe B. Rodgers, Colette J. Mustard, Ryan T. McLean, Sharon Hutchison, Antonia L. Pritchard

**Affiliations:** ^1^ 7709 Genetics and Immunology Department Division of Biomedical Research Institute of Health Research and Innovation University of the Highlands and Islands Inverness UK

**Keywords:** antibody, B‐cell, cancer, checkpoint inhibitor response, IgA, IgD, IgG, immunoglobulin, melanoma, tertiary lymphoid structure (TLS)

## Abstract

The B‐cell system plays an important role in the melanoma immune response; however, consensus has yet to be reached in many facets. Here, we comprehensively review human studies only, due to fundamental differences in the humoral response with animal models. Tumour‐infiltrating B‐cells are associated with contradictory prognostic values, reflecting a lack of agreement between studies on cell subset classification and differences in the markers used, particularly the common use of a single marker not differentiating multiple subsets. Tertiary lymphoid structures (TLS) organise T‐cells and B‐cells within tumours to generate a local anti‐tumour response and TLS presence associates with improved survival in response to immune checkpoint blockade, in late‐stage disease. Autoantibody production is increased in melanoma patients and has been proposed as biomarkers for diagnosis, prognosis and treatment/toxicity response; however, no consistent targets are yet identified. The function of antibodies in an anti‐tumour response is determined by its isotype and subclass; IgG_4_ is immune‐suppressive and robustly correlate with poor patient survival in melanoma. We conclude that the current B‐cell literature needs careful interpretation based on the methods used and that we need a consensus of markers to define B‐cells and associated lymphoid organs. Furthermore, future studies need to not only examine antibody targets, but also isotypes when considering functional roles.

## 1 INTRODUCTION

There are four major subtypes of melanoma, arising from melanocytes located in different body locations: cutaneous melanoma (CM), acral lentiginous melanoma (ALM), uveal melanoma (UM) and mucosal melanoma (MM). CM is associated with exposure to ultraviolet radiation (UVR), resulting in a higher tumour mutation burden (TMB) than UM, ALM and MM; conversely, CM has a lower level of chromosomal aberration compared with the other subtypes (Hayward et al., 2017). CM has been widely used as a model of immune‐oncology, due to its highly immunogenic and immune evasive capacities, linked to the high TMB providing diverse sources of ‘non‐self’ antigen recognised by the immune system (Hutchison & Pritchard, 2018). T‐cells have been particularly well studied, which has directly revolutionised cancer treatment, with immune checkpoint blockade (ICB) now prescribed as the gold standard of care for metastasised CM, significantly improving overall survival rates (reviewed by (Robert, 2020)). Not all patients respond to ICB and a further subgroup develop resistance; research into what drives these responses has prompted a recent resurgence of interest in B‐cells (Cabrita et al., 2020; Helmink et al., 2020; Petitprez et al., 2020).

This review will discuss the existing evidence of the nature of B‐cell responses in patients with melanoma and how this might influence response to ICB. This includes the seemingly contradictory observations of pro‐ and anti‐tumourigenic responses mediated by B‐cells, the effects of the tumour microenvironment, the development of tertiary lymphoid structures and how all observations are influenced by the B‐cell and tissue markers selected. We also discuss the potential to use antibodies as biomarkers for diagnosis, prognosis, therapeutic response or toxicity. We only focus on data derived from human studies, due to a lack of concordance of B‐cell subtypes with animal models. A contextualising overview of B‐cell antibody production can be found in Supplementary File 1 and Supplementary Figure 1, which provides an overview of the humoral immune system.

## 2 B‐CELL SUBTYPE IDENTIFICATION

An important consideration when reviewing the literature on tumour immune response is how the cells were characterised. CD19 and CD20 are the most commonly used markers to define the presence of B‐cells (e.g. Table 1 and Supplementary Table 1). They are expressed across all B‐cell subsets, except some types of plasma cells, and are commonly used as ‘pan B‐cell’ markers. CD19 expression can infer information on the activation status of the B‐cell and may help to distinguish short‐lived plasma cells from long‐lived plasma cells, compared with CD20. The main impact of only using a pan B‐cell marker like CD19 or CD20 is that it does not allow us to begin to work out the nature of this response, which can have anti‐tumourigenic or immunosuppressive (likely pro‐tumourigenic) effects.

**TABLE 1:** Markers used to define B‐cell subtypes in melanoma

B‐cell (subtype)	Method used	Markers used	Reference
B‐cell	Immunohistochemistry	CD20^+^	Balatoni et al. (2018), Bosisio et al. (2016), Cabrita et al. (2020), Cipponi et al. (2012), Erdag et al. (2012), Garg et al. (2016), Halse et al. (2018), Harlin et al. (2009), Helmink et al.( 2020), Hillen et al. (2008), Kotlan et al. (2019), Ladányi et al. (2011), Martinez‐Rodriguez et al. (2014), Messina et al. (2012), Meyer et al. (2012a), Somasundaram et al. (2017)
B‐cell	Immunohistochemistry	CD19^+^, CD20^+^	Amaria et al. (2018)
B‐cell	Mass cytometry	CD19+CD45+	(Helmink et al. (2020)
Circulating B‐cells	Flow cytometry	CD19^+^	Carpenter et al. (2009), Das et al. (2018)
Naïve B‐cells	Mass cytometry	CD19^+^CD45^+^ CD27^−^IgD^+^	Helmink et al. (2020)
Circulating naïve B‐cells	Flow cytometry	CD19^+^CD27^−^	Das et al. (2018)
Transitional B‐cell	Mass cytometry	CD19^+^CD45^+^CD24^++^CD38^++^CD10^+^CD27^−^IgD^+^	Helmink et al. (2020)
Transitional cell‐like	Immunohistochemistry	CD19^−^CD20^+^CD138^−^CD5^+^	Griss et al. (2019)
Circulating memory (non‐class‐switched) B‐cells	Flow cytometry	CD19^+^CD27^+^IgM^+^	Das et al. (2018)
Memory (non‐class‐switched) B‐cells	Mass cytometry	CD19^+^CD45^+^CD27^+^IgD^+^	Helmink et al. (2020)
Circulating memory (class‐switched) B‐cells	Flow cytometry	CD19^+^CD27^+^IgM^−^	Das et al. (2018)
Memory (class‐switched) B‐cells	Mass cytometry	CD45^+^CD19^+^CD27^+^IgD^−^	Helmink et al. (2020)
Plasma cell	Immunohistochemistry	CD138^+^	Bosisio et al. (2016), Cipponi et al. (2012), Erdag et al. (2012)
Plasma cell‐like	Immunohistochemistry	CD19^+^CD20^−^CD138^+^	Griss et al. (2019)
Plasma cell‐like	Mass cytometry	CD19^+^CD20^−^CD45^+^CD22^−^CD39^++^CD27^++^	Helmink et al. (2020)
Circulating plasmablasts	Flow cytometry	CD19^+^CD27^+^CD38^hi^	Das et al. (2018)
Circulating plasmablasts	Flow cytometry	CD19^+^CD27^bright^CD38^+^CD86^+^	Carpenter et al. (2009)
Circulating plasmablasts	Flow cytometry	CD19^+^CD20^low/−^CD38^high^CD27^+^CD3^−^CD14^−^IgA^−^IgM^−^	Defalco et al. (2018)
Plasmablast‐like	Immunohistochemistry	CD19^+^CD20^−^CD38^+^CD138^−^	Griss et al. (2019)
Memory B‐cell‐like	Immunohistochemistry	CD19^+^CD20^+^CD38^−^CD138^−^CD27^+^	Griss et al. (2019)
Germinal centre B‐cells	Immunohistochemistry	CD20, AID, Ki67	Cipponi et al. (2012)
Germinal centre B‐cell‐like	Immunohistochemistry	CD20^+^CD38^+^CD138^−^CD5^−^	Griss et al. (2019)
Germinal centre B‐cell‐like	Mass cytometry	CD19^+^CD20^++^CD38^+^CD27^−^IgD^−^CD86^+^CD95^+^	Helmink et al. (2020)
Activated B‐cell‐like	Immunohistochemistry	CD19^+^CD20^−^CD38^−^CD138^−^CD27^+^	Griss et al. (2019)

The markers for specific B‐cell subtypes are less well defined and have been inconsistently used between studies making it difficult to compare observations across the literature. This issue is clearly illustrated when examining the markers used to define different B‐cell subsets in the melanoma studies discussed within this review (Table 1 and Table 2).

**TABLE 2:** Markers used to describe a TLS in melanoma

Citation	Method used	Disease stage(s)	Structure being defined	Markers used
Griss et al. (2019)	Immunohistochemistry	Stage IV	Primary follicle‐like TLS	CD20^+^CD4^+^CXCL13^+^CD21^+^CD23^−^BCL6^−^DAPI
			Immature secondary follicle‐like TLS	CD20^+^CD4^+^CXCL13^+^CD21^+^CD23^+^BCL6^−^DAPI
			Mature secondary follicle‐like TLS	CD20^+^CD4^+^CXCL13^+^CD21^+^CD23^+^BCL6^+^DAPI
Helmink et al. (2020)	Immunohistochemistry	Undefined metastasis	Tertiary lymphoid structure	CD20, CD8, FOXP3, CD4, CD21 (DAPI or H&E dependent on application used)
		Lymph node metastases (on treatment)	Tertiary lymphoid structure	CD21, CD23, CD20, CD4, Syto13, MECA−79
		Subcutaneous mass (no treatment)	Tertiary lymphoid structure	CD20, CD21, CD23, H&E
Cipponi et al. (2012)	Immunohistochemistry	Metastatic	Ectopic lymphoid structures	CD20+ B‐cells
				CD20+CD21 (*data not shown*: VCAM and FDCag)
				CD20, CD138, CD21, Melan‐A, CD8, DC‐LAMP, PNAd, AID (*data not shown*: Ki67)
	Immunohistochemistry	Primary (stage I/II)	incomplete/early signs of ectopic lymphoid structures	CD20, CD21, PNAd, CD8, DC‐LAMP
Messina et al. (2012)	H&E staining	Metastatic	Ectopic lymph node‐like structures	H&E staining
	Immunohistochemistry			CD20, CD3, CD4, CD8, CD86. FOXP3
Cabrita et al. (2020)	Immunohistochemistry	Distant subcutaneous metastasis; Lung metastasis	Tertiary lymphoid structure	CD20, Ki67, SOX10, CD3, CD8
		A representative melanoma with a TLS		CD20, CD3, CD8, CD4
Garg et al. (2016)	Immunohistochemistry	Primary tumour	Tertiary lymphoid‐like structures	CD20+
Ladányi et al. (2007)	Immunohistochemistry	Primary tumour	Lymphocyte aggregates suggestive of lymphoid organ structure	CD1a, DC‐LAMP, CD45R0 (*data not shown*: CD25)
Ladányi et al. (2011)	Immunohistochemistry	Primary tumour	Follicle‐like aggregates of lymphocytes	CD20
				CD20 plus CD45R0 or CD25
Bosisio et al. (2016)	Immunohistochemistry	Primary tumour	Tertiary lymphoid structures	In CD138 and IgA‐positive tumours they stained for CD20, CD3, CD21, PNAd,
Martinet et al. (2012)	Immunohistochemistry	Primary tumour	HEV‐positive lymphocyte aggregates	CD3, CD20, CD8 and MECA−79

There is, therefore, a clear and urgent need for appropriate interpretation when pan B‐cell markers are used and a harmonisation of B‐cell subtype definition and identification (e.g. reviewed in (Sanz et al., 2019)). This is not only an issue in the context of melanoma, but as illustrated from the data gathered in Supplementary Table 1, the most serious impact is the inability to bring together data from across the solid tumour field to fully understand what influences the role and impact of B‐cells.

## 3 B‐CELLS AND CANCER

Despite the limitations outlined above, there is no question that B‐cells form part of the immunogenic response to CM and other cancers (e.g. Table 1, Supplementary Table 1; reviewed in (Mukherji, 2013, Fridman et al., 2020)).

The B‐cell responses or antibody specificities detected in cancer patient serum, lymph nodes and tumours include: (i) low‐affinity IgM, possibly autoantibodies produced by innate‐like B‐cells (Zhang, 2013); (ii) matured (undergone somatic hypermutated (SHM) and class switch recombination (CSR)) autoantibodies, recognising native self‐proteins (e.g. (Kijanka et al., 2010; Li et al., 2020)); (iii) cancer‐antigen immune responses (IgM, IgA and IgG), which can be against well‐described tumour antigen, including antigens of lineage‐specific differentiation (e.g. (Fishman et al., 1997; Sahin et al., 1995)), cancer‐testis (CT) antigens (e.g. (Chen et al., 1997; Jager et al., 1998; Sahin et al., 1995)), mutational neoantigens (e.g. (Scanlan et al., 1998; Yasuda et al., 2002)) and viral sources (e.g. (Louis et al., 2010; Meng et al., 2018; Smith et al., 2020)); (iv) non‐specific activation of B‐cells in a sufficiently inflammatory environment, including cells putatively labelled as regulatory B‐cells (B_reg_; reviewed in (Sarvaria et al., 2017)); (v) CD86^hi^CD21^lo^ antigen‐presenting B‐cells in the TLS, capable of stimulating T‐cell activation when assessed in vitro (Wennhold et al., 2021).

Furthermore, the associations of B‐cells and/or antibodies present in patients with cancer can have anti‐ or pro‐tumourigenic effects, which can only be identified from careful characterisation, as will be outlined in the context of melanoma within this review.

## 4 B‐CELLS INFILTRATING THE MELANOMAS

Although reports are highly variable, on average ~30–50% of tumour‐infiltrating lymphocytes (TILs) in CM can be B‐cells; this does not occur uniformly in each metastasis within an individual, or in tumours between different individuals. These inconsistencies could be due to differences in markers used to define a B‐cell (Table 1 and Supplementary Table 1), the sensitivity of the identification method used, melanoma stage, how representative of the total tumour the examined tissue section is (Erdag et al., 2012) and the location of the tumour (e.g. higher densities of CD20^+^ B‐cells in lymph node metastases vs. subcutaneous metastases (Balatoni et al., 2018)). Analysing whole tumour sections, it was found that approximately 77% of primary melanoma tumours contained CD20^+^ B‐cells. Using a metastatic melanoma core tissue array, it was shown that 35% of tumours contained CD20+ B‐cells (Somasundaram et al., 2017). Despite these caveats, significantly decreased B‐cell numbers are consistently found in advanced stage IV melanoma compared with earlier stages (Erdag et al., 2012; Garg et al., 2016; Somasundaram et al., 2017) and those that are there in late‐stage disease are frequently a suppressive phenotype (increased expression of *IL*‐*10* and *TGFB1*), which co‐occur with increased B‐cell exhaustion marker expression (*PDCD1*, *FCRL4*, *SIGLEC6* and *CD22*) (Griss et al., 2019).

### 4.1 Location and organisation of TILS

**FIGURE 1:**
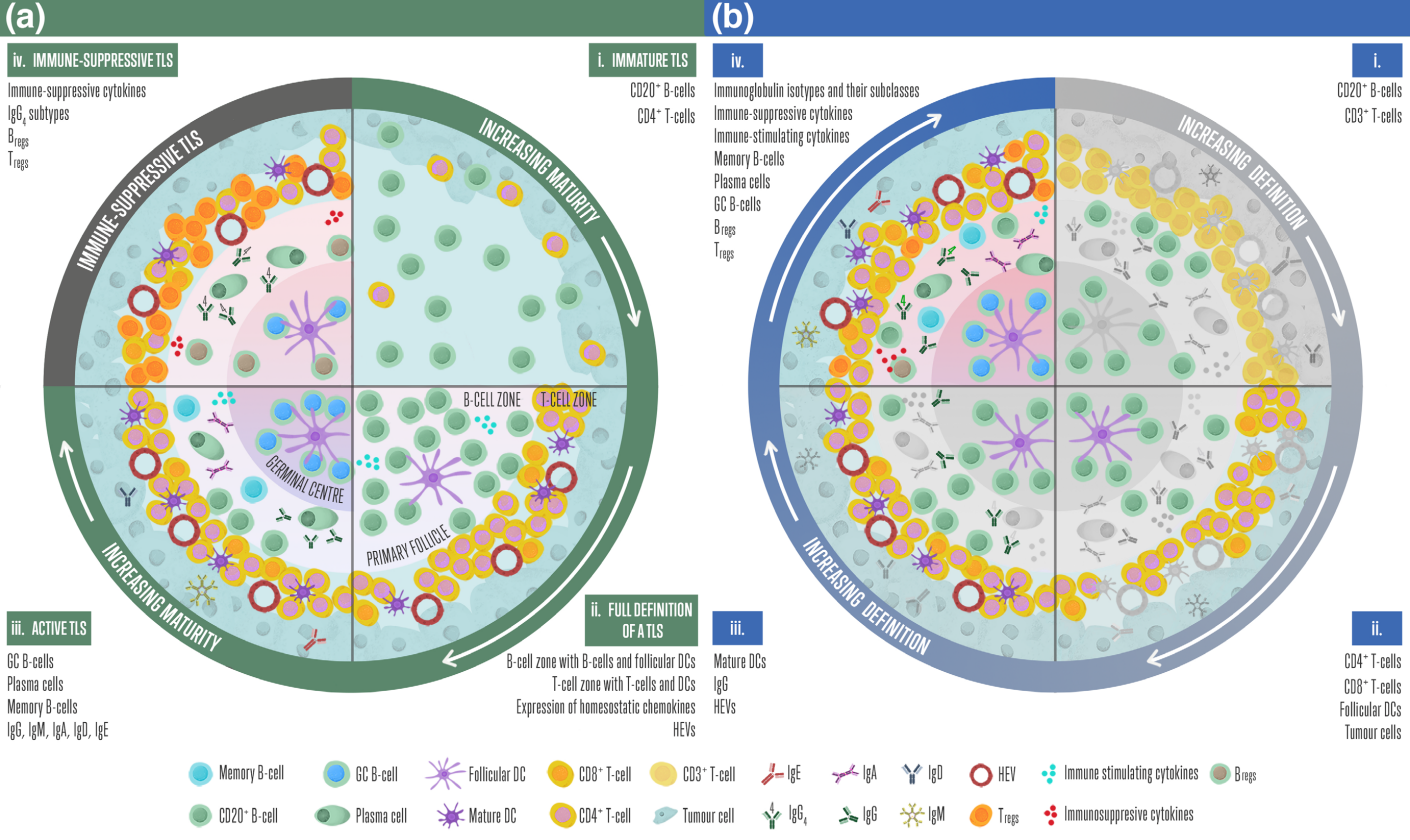
Defining a tertiary lymphoid structure. (a) As TLS mature, they become more organised structures. Starting from the top right‐hand quadrant (i) An immature TLS, with B‐cells and T‐cells infiltrating the tumour but have not yet organised into discrete zones. (ii) A full definition of a TLS is shown, with expression of homeostatic chemokines and the presence of HEVs, a clear B‐cell zone with the primary follicle containing B‐cells and follicular DC, and a T‐cell zone with T‐cells and DCs. (iii) An active TLS with a secondary follicle formed where B‐cells undergo clonal expansion and affinity maturation to elicit an *in situ* adaptive immune response. (iv) The hypothetical immune‐suppressive TLS that may occur after extended exposure to antigen, featuring immune‐suppressive cytokines, IgG_4_, B_regs_ and T_regs_ and the structures beginning to resolve. (b) All four quadrants contain the same types of cells, antibodies, cytokines and tissue structures, but by increasing the number of cell and tissue markers a more detailed profile of a TLS will be achieved. (i) If single markers are used to detect B‐cells and T‐cells (e.g. CD20^+^ and CD3^+^, respectively), cell subtype information will be lost. (ii) A better‐defined TLS, with CD8^+^ and CD4^+^ differentiation between T‐cell subtypes and markers for follicular DC and tumour cells. (iii) A TLS, as defined by Dieu‐Nosjean et al. (2014), using markers for B‐cells, follicular DC, CD8^+^ and CD4^+^ T‐cells and HEVs. IgG is the most common considered antibody isotype. (iv) Further definition revealing the active or immune‐suppressive status of the TLS, including active B‐cell subtypes, B_reg_, T_reg_, immunoglobulin isotypes and subclasses, HEVs, immune‐stimulating or immune‐suppressive cytokines, mature DC and follicular DC

TILs are not confined to any one location within a tumour, although their location, either intratumoural, stromal or marginal, can be prognostic (Antohe et al., 2019; Barnes & Amir, 2017; Fu et al., 2019; Mihm & Mulé, 2015; Peled et al., 2019). When comparing studies on TILs, it is important to consider the context of the tumour section(s) used. For example, tissue cores only provide information about a limited tumour area, while whole tissue sections provide a larger field of investigation but often lose key positional information (Hendry et al., 2017a). Infiltrating B‐cells are often associated with TLS (Table 2) in various stages of formation, during which they become increasingly organised, resembling structures found in the secondary lymphoid structure (SLS) lymph nodes (Figure 1), likely enabling localised tumour‐specific antigen presentation to T‐cells and antibody production ((Wennhold et al., 2021) and reviewed by (Lauss et al., 2021)).

Continuing the theme in this review on the importance of consensus on B‐cell classifications and markers used to identify them, the histopathological investigations of immune cell location and organisation within tumours (including TLS) have been historically inconsistent between studies, making comparisons and cohesive conclusions difficult. In a two‐part review, Hendry et al. (2017) proposed a standardised method to evaluate TILs in solid tumours to tackle this issue (Hendry et al., 2017a, 2017b). Once consensus has been reached, improved spatial‐omics technologies (Lewis et al., 2021) can be applied, to better understand the location and organisation of TIL within the tumour.

## 5 PHENOTYPE OF B‐CELLS INFILTRATING MELANOMA

The composition of the melanoma‐associated B‐cell subtypes dictates the type, efficacy and direction of response. The main techniques used to phenotype B‐cells are immunohistochemical staining or the flow, or mass, cytometry of TILs, suggesting a range of B‐cell subtypes can be present in melanomas (Table 1). Based on these user‐defined phenotypes, plasmablasts, plasma cells, activated B‐cells, germinal centre B‐cells, transitional B‐cells and memory B‐cells have been identified in melanomas (Table 1). Since multiple B‐cell subtypes are present in melanoma, this suggests that different B‐cell functionalities are at play in the immune response. For example, plasmablasts and plasma cells are active antibody‐secreting cells and their presence in melanomas could indicate tumour antigen recognition by antibodies are present. Further classification of the infiltrating memory B‐cells has not been performed, so it is unknown if they are affinity‐matured products of a germinal centre reaction (IgG^+^ and undergone SHM) or derived from germinal centre independent mechanisms (IgM^+^ and not undergone SHM); but are likely to function either as plasmablast precursors or produce germinal centre B‐cells (Pape & Jenkins, 2018). Moreover, the different levels of maturity present across infiltrating B‐cell subsets indicate differences in B‐cell receptor (BCR) affinity to antigen are present, which influences the ability to produce a strong targeted response. The lack of consistently agreed upon B‐cell subtyping categories and definitions (Table 1) has, however, hampered consensus in reported studies, and therefore, incomplete B‐cell functions in response to melanoma have likely been reported (Amaria et al., 2018; Bosisio et al., 2016; Cabrita et al., 2020; Carpenter et al., 2009; Cipponi et al., 2012; Das et al., 2018; Erdag et al., 2012; Garg et al., 2016; Griss et al., 2019; Harlin et al., 2009; Helmink et al., 2020; Hillen et al., 2008; Kotlan et al., 2019; Ladányi et al., 2011; Martinez‐Rodriguez et al., 2014; Messina et al., 2012; Meyer et al., 2012a; Somasundaram et al., 2017); Table 1, Supplementary Table 1. The consistent use of a single marker (usually CD19 or CD20, or the expression of Ig) to identify the presence of B‐cells has particularly resulted in inconsistent interpretation, as more information is required to understand whether they have a pro‐ or anti‐ tumourigenic role (Table 1; Supplementary Table 1).

Single‐cell sequencing (scRNA‐seq) has been used to perform an in‐depth analysis of the T‐cells present within CM and UM (e.g. (Durante et al., 2020; Tirosh et al., 2016; Yan et al., 2021)) but an equivalent comprehensive analysis of B‐cells using this technology has not been completed, to date. One recent study used k‐means clustering of scRNA‐seq data to split B‐cell TILs into four clusters, characterised by user‐defined phenotypes based on markers (1) switched, activated IgD^−^; (2) plasma cells; (3) unswitched IgD^+^ and (4) switched, activated IgD^−^, with unique markers relative to (1) (Helmink et al., 2020), while another used gene sets to identify the presence of activated, immature and memory B‐cells and more rarely, plasma cells in melanomas (Cabrita et al., 2020).

## 6 MICROENVIRONMENT FACTORS INFLUENCING B‐CELL INFILTRATION AND PHENOTYPES

The mechanisms controlling B‐cell infiltration are not fully understood but are likely influenced by the expression of chemokines in the tumour microenvironment, which can influence the trafficking and phenotype of lymphocytes in the tumour microenvironment.

In mice, CXCL13 is vital for the development of TLS (Ansel et al., 2000) and this has been supported in humans, with intratumoural expression of *CXCL13* correlating with increased B‐cell infiltration (Bindea et al., 2013; Gu‐Trantien et al., 2013; Harlin et al., 2009; Workel et al., 2019), and co‐expression with its receptor CXCR5 on the same structure indicating the presence of TLS (Cabrita et al., 2020). Further, circulating CXCL13 levels are increased in melanoma patients after treatment with ICB (Das et al., 2018), potentially linking TLS with survival (as detailed below). Importantly, however, upon activation by CXCL13, CXCR5 can induce proliferation (Legler et al., 1998) via pathways frequently modulated within CM, including signalling through MAPK, PI3K and RAC1 (Hayward et al., 2017). Although not yet explored in melanoma, this cytokine axis was associated with progressive metastatic disease and poor survival, in colorectal cancer (Zhu et al., 2015), breast cancer (Panse et al., 2008) (that is not HER2^+^ (Razis et al., 2012)) and prostate cancer (El‐Haibi et al., 2011; Singh, Singh, Sharma, et al., 2009; Singh, Singh, Singh, et al., 2009). Therefore, while CXCL13 in the tumour microenvironment might indicate B‐cell infiltration, tumour CXCR5 expression (rather than the TLS) could have a pro‐tumourigenic effect.

The presence of a TLS is, however, not the only factor that influences the phenotype of B‐cells infiltrating a melanoma. It has been hypothesised various B‐cells subtypes can develop regulatory properties to become B_regs_, dependent on tumour microenvironmental cues (Largeot et al., 2019; Michaud et al., 2020). There has been more robust evidence of B_regs_ in mice; however, translation into humans has been impeded by lack of equivalent human markers (Lighaam et al., 2018). Evidence to support B_reg_ development in humans came from treating human peripheral B‐cells with ‘breast cancer cell‐conditioned’ media to create tumour‐evoked B‐cells with B_reg_ features (Olkhanud et al., 2011). Furthermore, evidence in melanoma suggests some TIL with plasmablast‐like marker phenotype also have B_reg_ features (Griss et al., 2019). B_regs_ are believed to play an immunosuppressive role in the tumour microenvironment through the production of specific cytokines (e.g. IL‐10, IL‐35 and TGF‐β). Measurement of the immunosuppressive cytokines in the microenvironment is not a reliable measure of B_regs_ as they are not the only source(s) of these cytokines, which could include melanomas themselves, regulatory T‐cells (T_regs_) and dendritic cells (DC). These issues have meant that the impact of B_regs_ on immune control in melanoma has yet to be elucidated, and consequently there is a broad scope for research in this area.

## 7 PROGNOSTIC ROLE OF B‐CELLS INFILTRATING MELANOMA

Numerous studies have investigated the association between tumour infiltrating B‐cells and progression‐free survival or overall survival in CM, but the findings have been inconclusive to date. Using whole tumour sections, a positive correlation between B‐cell presence and (i) overall survival in primary CM (Garg et al., 2016; Ladányi et al., 2011); (ii) decreased metastatic progression in primary CM (Ladányi et al., 2011) and (iii) increased survival time in patients with metastasised CM (Erdag et al., 2012) have been reported. Furthermore, high expression of *CD19*, *CD20* or B‐cell signatures in CM data from the Cancer Genome Atlas (TCGA) has been correlated with significantly improved survival (Garg et al., 2016; Helmink et al., 2020; Iglesia et al., 2016). In contrast, one study found no correlations between B‐cell infiltration and survival (Hillen et al., 2008) and other studies have shown association between B‐cells and poor prognosis in primary CM (Martinez‐Rodriguez et al., 2014; Meyer et al., 2012b). These inconsistent data are likely confounded by the differences in definitions of B‐cells used between studies (Table 1) and studies using precise phenotyping of the B‐cells subtypes and identification of the antibody isotypes present are now required.

There have also been investigations of the association between the humoral response and prognosis association with therapeutic intervention. Evidence of acquired resistance to BRAF and MEK inhibitors induced by tumour‐associated CD20^+^ B‐cells came from an in vitro analysis, which suggested this was mediated by B‐cell production of growth factor IGF‐1 (Somasundaram et al., 2017). Further, this study revealed potential clinical application in a small pilot trial of B‐cell depletion by the anti‐CD20 antibody ofatumumab (NCT01376713) in a cohort of CM patients (*n* = 10). Evidence of clinical activity was found in 8/10 patients, and a benefit in measurable disease was found in 6/10 patients though overall response evaluation showed progressive disease in the majority of patients (Somasundaram et al., 2017). High T‐cell/low B‐cell gene expression signatures in pre‐treatment tumour samples from patients subsequently treated with BRAF and MEK inhibitors were associated with longer survival compared with high T‐cell/high B‐cell signatures (Brase et al., 2021). Neither of these studies investigated the subtypes of B‐cells involved, whether they were an immunosuppressive phenotype, or if they were associated with TLS within the tumour. This latter point is of particular interest due to recent work that has correlated TIL associated with TLS with good response to ICB, which will be further detailed in the section specifically focussed on the TLS.

## 8 CHARACTERISTICS OF THE BCR OF TUMOUR‐INFILTRATING B‐CELLS

Assessment of the BCR expressed by the B‐cell TIL can provide vital information about the characteristics of the infiltrating cells, including whether they have undergone Ig isotype switching, the isotypes produced and the clonality of the B‐cells present, which can be used to infer the quality, context and direction of the B‐cell response. It is, therefore, vital that both the immunoglobulin isotype and subclass are measured, as it is not possible to deduce the immunosuppressive or anti‐tumour impact of the cell present or of the antibodies produced without this assessment (Supplementary Figure 1).

Bulk tumour gene expression analysis showed the BCR heavy chain isotype expression was, in order of abundance, IgG>IgA>IgM>IgD, measured in the TCGA SKCM RNA‐seq data (Cancer Genome Atlas, 2015; Selitsky et al., 2019); these samples spanned all stages of CM disease and outcomes. Further analysis has indicated that increased ratios of IgA, IgD or IgE to total Ig were associated with a negative prognosis in CM (Bolotin et al., 2017). Similarly, earlier studies showed by mRNA expression array that IgG and IgM heavy chain genes, and both κ and λ light chain genes, were expressed in melanoma biopsies (Harlin et al., 2009) and that plasma cells in CM primaries were predominantly IgG^+^ or IgA^+^ (Bosisio et al., 2016). These studies were unable to distinguish Ig subclasses, but this has been specifically examined by other investigators, showing that BCR tends to have switched towards the IgA_1_, IgA_2_, IgG_1_ and IgG_2_ subclasses when they have infiltrated melanoma TLS (Cipponi et al., 2012). Importantly, BCR diversity was lower (i.e. increased clonality) and SHM was higher in primary tumour and regional subcutaneous metastases compared with distant metastases (Selitsky et al., 2019) and decreased BCR diversity was associated with better survival in melanoma (Bolotin et al., 2017; Iglesia et al., 2016; Selitsky et al., 2019). Within a clonally expanded B‐cell cluster, multiple antibody subclasses can be found suggesting that CSR occurs again after clonal expansion. Indeed, individuals with higher levels of B‐cell IgG_3_–IgG_1_ switches, likely indicating B‐cell progression in the face of persistence of antigen (Jackson et al., 2014), had significantly better clinical outcomes in melanoma (Hu et al., 2019).

## 9 TERTIARY LYMPHOID STRUCTURES

The role of the TLS in cancer has recently come under increased scrutiny, after associations with ICB response were identified (reviewed by (Lauss et al., 2021)). TLS display remarkable plasticity, providing a temporary system to elicit an in situ adaptive immune response, resolving once the source of the antigen or inflammation has been eradicated (Wotherspoon et al., 1993). When fully matured, TLS is highly organised structures bearing a strong resemblance to SLS such as lymph nodes; Figure 1a. As with other aspects of the B‐cell response, historically there has been inconsistency in the identification criteria for a TLS, which has likely contributed to the ambiguous prognostic value in cancer described in previous studies. To provide consensus, Dieu‐Nosjean et al. (2014) proposed a set of criteria to define a TLS, which includes a) the manifestation of immune cells into two discrete areas; b) a B‐cell zone containing naïve B‐cells and follicular DCs, c) a T‐cell zone containing T‐cells and DCs; d) the presence of structurally distinct blood vessels, known as high endothelial venules (HEVs), which allow the trafficking of lymphocytes into lymphoid tissues and areas of chronic inflammation and e) expression of homeostatic chemokines including CXCL13 (Dieu‐Nosjean et al., 2014). The addition of successive information provides increasing detail, important for interpretation of the data (e.g. as illustrated in Figure 1).

## 10 PRESENCE OF TLS IN MELANOMA

The first evidence of TLS formation in melanoma was circa 2011, described as ‘follicle‐like aggregates’ or ‘ectopic lymphoid structures’ in ~25% of primary and metastasised CM (Cipponi et al., 2012; Ladányi et al., 2011). A 12 chemokine gene expression signature (consisting of *CCL2*, *CCL3*, *CCL4*, *CCL5*, *CCL8*, *CCL18*, *CCL19*, *CCL21*, *CXCL9*, *CXCL10*, *CXCL11* and *CXCL13*) has been suggested to predict the presence of TLS via indication of co‐presence of CD20^+^ B‐cells, CD86^+^ DC and CD4^+^FoxP3^−^ and CD8^+^ T‐cells in stage IV CM (Messina et al., 2012). Now, it is well established melanomas can have multiple TLS present, which can be at varying stages of maturity; Table 2, Figure 1a. This is independent of the tumour mutational burden, indicating TLS formation is not dependent on immune response due to neoantigens (Cabrita et al., 2020), unlike the CD8^+^ T‐cell response in CM (Hutchison & Pritchard, 2018). The presence of TLS has shown contradictory associations with patient outcome (Cabrita et al., 2020; Castino et al., 2016; Dieu‐Nosjean et al., 2014; Figenschau et al., 2015; Germain et al., 2014; Martinet et al., 2011; Yamaguchi et al., 2020); reviewed by (Fridman et al., 2020; Lin et al., 2019), which were likely confounded by the definitions of TLS used (Table 2). Since consistent criteria to identify a TLS have been proposed (Dieu‐Nosjean et al., 2014), studies have more consistently shown a positive outcome associated with TLS. Though, it is important to note that even when the identification criteria were met, due to methodological limitations this needed to be done over consecutive assays on different histology sections (Table 2). The development of conjugated antibodies that can be released from sections, allowing large‐scale multiplexing immunohistochemistry (e.g. Miltenyi REAlease^®^ antibodies) will improve the consistency of reporting TLS and other aspects of the tumour architecture.

## 11 COMPOSITION OF TLS IN MELANOMA

While ~50% of metastatic CM were positive for CD20^+^ B‐cell infiltrates, only ~25% were associated with features resembling follicles of a TLS (Cipponi et al., 2012). Significant focus has been on demonstrating associations between the different types of cells and the structures within tumours, to investigate TLS functionality. In fully formed TLS, mature follicular DC is associated with activated B‐cells and mature DC is associated with activated T‐cells, resembling the clusters of lymphocytes seen in SLS (Figure 1a). The composition of the TLS can provide vital information on the status of the B‐cell response; however, the interpretation of these data requires consideration of the markers used for definition (Figure 1b; Table 2).

HEVs are found near TLS but not elsewhere in metastatic CM sections (Cipponi et al., 2012). Mature DC, CD3^+^CD8^+^ T‐cells and CD20^+^ B‐cells specifically localise in HEV‐rich areas, suggesting they play a key role in transporting these cells into the tumour microenvironment; conversely, T_reg_ infiltration is not enhanced in the tumour by HEV (Martinet et al., 2012). A higher HEV density is associated with better clinical markers of melanoma such as low Breslow thickness, early disease staging, low levels of tumour invasion and tumours with a high lymphocytic infiltration (Avram et al., 2013; Martinet et al., 2011, 2012).

A proportion of single B‐cells infiltrating CM express AID and BCL6 required for the formation of germinal centres (Cabrita et al., 2020); furthermore, B‐cell follicles with nucleic expression of AID are often positive for Ki67 and in close contact with melanoma, indicating cell proliferation in response to tumour is occurring (Cipponi et al., 2012). Activated T‐cells of various subtypes are increased within melanoma TLS and are often found in close contact with CD20^+^ B‐cells (Cipponi et al., 2012; Helmink et al., 2020; Ladányi et al., 2007, 2011), including CD40L^+^ activated T‐helper (T_H_) cells (Cabrita et al., 2020), suggesting T‐cell interaction with antigen‐presenting cells and B‐cells with T_H_‐cells in the TLS, leading to the activation of both cell types.

While B‐cell activation and maturation can occur in TLS, terminal differentiation to plasma cells seems to be a rare occurrence. In invasive primary CM or ALM, only a small proportion contained CD138^+^ plasma cells (~6%), and of these, ~12% were associated with the TLS (Bosisio et al., 2016). These plasma cells were often IgA^+^, suggesting recirculation of plasma/plasmablast cells from the skin to lymph nodes, rather than in situ production (Bosisio et al., 2016). In melanoma metastases, plasma cells are often found surrounding TLS in an irregular and diffuse pattern but are also rare within TLS (Cipponi et al., 2012). Furthermore, the presence of plasma cells has been associated with a favourable prognosis in some solid tumours (Berntsson et al., 2016; Gentles et al., 2015), including metastatic melanoma (Erdag et al., 2012); however, in primary CM, infiltration by IgA^+^CD138^+^ plasma cells is associated with markers of negative prognosis, including high Breslow thickness, high mitotic rate and the presence of ulceration (Bosisio et al., 2016). A further two early studies also showed an association with a poor prognosis in CM; however, their method of identifying plasma cells was not explicitly stated (Mascaro et al., 1987; Weissmann et al., 1984).

## 12 PROGNOSTIC VALUE OF TLS IN MELANOMA

It is also important to consider that the TLS is dynamic, with the presence and maturity of TLS potentially altered by treatment. Patients experiencing an active, tumour clearing, immune response tended to have mature TLS, compared with patients who did not respond to anti‐CTLA4 and anti‐PD1 ICB immunotherapies (e.g. (Cabrita et al., 2020; Helmink et al., 2020; Ladányi et al., 2007; Riaz et al., 2017)). Switched memory B‐cells and increased BCR diversity are also significantly enriched in tumours from patients who responded to ICB (Helmink et al., 2020), and some therapies enhance plasma cell TLS differentiation (Soiffer et al., 1998). High density of activated and co‐localised T‐cells, CD20^+^ B‐cells and DC populations are also associated with improved survival (Cabrita et al., 2020; Helmink et al., 2020; Ladányi et al., 2011). While the presence of FOXP3^+^ T_reg_ in TLS has not yet been described in the context of CM survival, they have been associated with poorer patient outcome in other cancers (Gobert et al., 2009; Joshi et al., 2015). Of note, germinal centre‐like B‐cells (CD19^+^CD20^+^CD38^+^CD27^−^IgD^−^CD86^+^CD95^+^) were found in melanomas regardless of their response to immunotherapy, suggesting their presence is not necessarily indicative of mature TLS (Helmink et al., 2020).

Notably, changes in circulating B‐cell populations also occur due to treatment, likely at least in part as a function of TLS activity. Patients on ICB combination therapy compared with monotherapy showed a modest increase in circulating class‐switched memory B‐cells, CD21^lo^ B‐cells and plasmablasts (Das et al., 2018). Similarly, patients with metastatic but non‐progressing melanoma treated with anti‐CTLA4 had higher levels of circulating plasmablasts compared with healthy controls (Defalco et al., 2018).

## 13 ANTIBODIES AS BIOMARKERS

Currently, the measurement of circulating lactate dehydrogenase (LDH) levels is the only serum marker in clinical use for melanoma staging (AJCC 8th edition (Keung & Gershenwald, 2018)). Elevated serum LDH levels correlate with a poor prognosis (e.g. (Eton et al., 1998)) and can be used to predict and monitor response to treatment (e.g. (Diem et al., 2016)). Care needs to be taken when investigating possible serological biomarkers to ensure that the potential for false‐positive results is minimal, such as using healthy individuals and patients with other types of cancer as control groups (Zornig et al., 2015). Autoantibodies have potential for use as cancer biomarkers because they are present in the circulation, early in disease before clinical symptoms of disease appear (Disis et al., 1997; Trivers et al., 1995, 1996; Yao et al., 2012; Zhong et al., 2006) and are relatively stable in circulation for a long time (Neiman et al., 2019). An important perspective not often assessed in melanoma, however, is the longitudinal stability of antibody repertoires, particularly with changing tumour burden as disease progresses/with treatment (e.g. as assessed in lung adenocarcinoma (Li et al., 2020)).

## 14 ANTIBODIES AS BIOMARKERS FOR THE PRESENCE OF MELANOMA

To date, no consistent autoantibody targets for a biomarker have been identified in all patients and no panel has been sufficiently accurate for clinical use to detect melanoma, as summarised in Supplementary Table 2 (Karagiannis et al., 2015; Litvak et al., 2004; Zaenker et al., 2018; Zornig et al., 2015). The ability to perform high‐throughput proteome screens (e.g. (Gowen et al., 2018; Kijanka & Murphy, 2009)) opens the potential to identify novel melanoma‐specific antibody biomarker panels and characterise longitudinal changes associated with the presence of melanoma. Future studies must be validated using large cohorts, including other cancers, pathologies and healthy controls to find truly melanoma‐specific biomarkers.

## 15 ANTIBODIES AS PREDICTORS OF DISEASE OUTCOME

A reduction in antibody responses has been observed in patients with stage III/IV disease compared to those with stage I/II local disease (Gilbert et al., 2011). The reason for this has yet to be elucidated, but it could suggest that immune tolerogenic mechanisms switch off tumour‐associated responses after long‐term exposure to tumour antigen. The presence of circulating antibodies against melanoma‐associated antigens has been correlated with improved survival in some studies (e.g. (Fassler et al., 2019; Jones et al., 1981; Yuan et al., 2011)), but not in others (e.g. (Zornig et al., 2015)). IgG_4_ titres positively correlate with disease progression, indicating B‐cells in metastatic melanoma could have class‐switched to this inhibitory IgG subclass, which decreases binding of the other IgG subclasses (as discussed in more detail below) (Karagiannis et al., 2013). Reproducibility is likely hampered by the lack of studies examining Ig isotypes and their subclasses, and future studies should take this into consideration.

## 16 ANTIBODIES AS MARKERS OF RESPONSE TO THERAPY

While the appearance of autoantibodies and clinical signs of autoimmunity were strongly associated with improved relapse‐free and overall survival in one study of patients receiving adjuvant therapy with high‐dose interferon‐α_2b_ for stage II/III melanoma (Gogas et al., 2006), further studies did not replicate these observations (Bouwhuis et al., 2009, 2010). In contrast, more consistent findings have been shown in responders to ICB. These include elevated levels of autoantibodies in circulation against four melanoma differentiation antigens (gp100, MelanA/MART1, TRP1/TYRP1 and TRP2/TYPR2) and the CT antigen NY‐ESO‐1 (Fassler et al., 2019; Haag et al., 2018), which have been proposed as a method to track ICB response. The subclasses of antibodies that responded to these antigens have also been investigated in a few studies. In one, NY‐ESO‐1 and TRP1 IgG_1_ and IgG_2_, and TRP2 IgG_2_ were statistically significantly (*p *< .05) associated with positive response to checkpoint inhibition while IgG_3_ and IgG_4_ to the tested tumour antigens were similar in responder and non‐responders (Fassler et al., 2019). Another found that ICB responders had higher pre‐treatment IgG_2_ levels compared with non‐responders, but no differences in total IgG or other subclasses (Diem et al., 2019). Of note, total IgG and IgG against common viral epitopes in Epstein–Barr virus remained stable 6–9 weeks after initiation of ICB in both responders and non‐responders, indicating stability in established memory B‐cell responses during treatment (Fassler et al., 2019).

## 17 ANTIBODIES AS MARKERS OF IMMUNE‐RELATED ADVERSE EVENTS

Some patients on ICB experience serious immune‐related adverse events (irAEs), particularly with combination therapies (reviewed in (Robert, 2020)); antibodies have been suggested as biomarkers for predicting irAEs. One proteomic microarray study reported a change in the level of circulating autoantibodies was predictive of irAE risk in CM patients receiving a combination of two ICB compared to those receiving monotherapy (Gowen et al., 2018). Furthermore, this study suggested a potential causative role for autoantibodies in irAE development, with significant enrichment in antigen targets highly expressed in organs affected by irAEs or involved in pathways associated with immune pathology (Gowen et al., 2018). One study found no significant association between pre‐ipilimumab treatment reactivity to 29 common clinical autoantibodies and the development of irAEs (De Moel et al., 2019), while another suggested a peak in autoantibodies preceding serious irAE onset in response to BCG and ipilimumab, assessed by protein microarray, in patients with metastatic CM (Da Gama Duarte et al., 2018). The patient number was too small (*n* = 2) to make any definitive conclusions in this second study but illustrates that autoantibodies would perhaps be better analysed using a high‐throughput method, rather than using candidate proteins.

## 18 ANTIGEN AND EPITOPES RECOGNISED BY ANTIBODIES IN MELANOMA PATIENTS

In B‐cell cultures, ~28% of those derived from patients produced antibodies that recognised melanoma cells, compared to 2% of cultures derived from healthy controls (Gilbert et al., 2011); however, the identification of melanoma‐specific antigens has not been a hugely successful process. A large source of this lack of progress is that the epitopes recognised by B‐cells are rarely continuous linear peptides (in contrast to T‐cell epitopes); instead, the majority are conformational and therefore discontinuous sequences (Rubinstein et al., 2008; Sivalingam & Shepherd, 2012). This provides a significant challenge when identifying the epitopes recognised and the rarer linear sequences have been disproportionally mapped (Vita et al., 2019). Techniques such as protein arrays have enabled screening for the protein antigens recognised by antibodies, but do not provide any information on the epitope sequences recognised (e.g. review of the technology (Kijanka & Murphy, 2009); in melanoma (Gowen et al., 2018); in colorectal cancer (Kijanka et al., 2010)). These arrays tend to identify autoantibodies against common native proteins but can be modified to ensure they include targets of interest, such as tumour‐associated antigens and post‐translationally modified (PTM) proteins, which can aberrantly occur in malignantly transformed cells. For example, a classic T‐cell epitope (YMDGTMSQV) formed by PTM of tyrosinase specifically occurs in melanoma (Skipper et al., 1996) and alteration in glycosylation patterns seen in melanoma (Laidler et al., 2005) can increase the number of targets for tumour‐specific antibodies (Kotlan et al., 2019). Studies do not often consider the PTM of proteins, and therefore, there is considerable potential in this approach to identify previously unappreciated targets. Further, these arrays would not recognise epitopes created from tumour‐specific mutations, which in cancers such as melanoma, are an important source of immunogenicity (Hutchison & Pritchard, 2018).

Importantly, the initial epitope recognised by the adaptive immune system can be expanded to recognise multiple epitopes on an individual molecule, known as epitope spreading (reviewed by (Cornaby et al., 2015)). B‐cell epitope spreading has yet to be shown in melanoma; however, T‐cell epitope spreading following immunotherapy has been demonstrated (Chapuis et al., 2016; Corbière et al., 2011). B‐cell epitope spreading likely occurs simultaneously with their role as antigen‐presenting cells, where their BCR binds antigen from lysed tumour, processes and presents novel epitopes cells to T‐cells, which in turn become activated by T_H_‐cells of linked specificity.

## 19 B‐CELL RESPONSES ENCOURAGING TUMOUR IMMUNE EVASION

As with the T_reg_ axis attenuating the T‐cell response, there are processes in place to control the B‐cell response. One such mechanism is the production of IgG_4_, which is the least abundant of the IgG subclasses (~4% of the total) and has the highest affinity for the inhibitory receptor FcγRIIb. IgG_4_ can become hetero‐bivalent, meaning it cannot cross‐link antigens or form immune complexes like the IgG_1_ – IgG_3_, further hampering its immune‐activating capabilities (van der Neut Kolfschoten et al., 2007). IgG_4_ has, therefore, been hypothesised to exert an anti‐inflammatory effect, dampening FcR‐mediated processes in the presence of persistent antigen. It has been suggested that there is an evolution of the qualities of the antibody response over the progression of cancer, such that IgG_4_ levels rise after prolonged antigen exposure from the tumour (Aalberse et al., 1983; Jackson et al., 2014).

Certainly, in melanoma patients, IgG titres isolated from blood, lymph nodes, or TIL all exhibited higher proportional IgG_4_ subclass compared with skin or blood from healthy individuals (Karagiannis et al., 2013; Saul et al., 2016). Further, the presence of IgG_4_ correlates with poor patient prognosis in several tumour types (reviewed in (Crescioli et al., 2016)), and in agreement, elevated IgG_4_ titres have been found in metastatic melanoma patients, correlating with poor patient survival (Daveau et al., 1977; Karagiannis et al., 2013). Elevated tumour IgG_4_^+^ B‐cell infiltrate and serum IgG_4_ levels are also associated with an increased risk of melanoma progression from early stages, indicating immunosuppressive mechanisms may be present and active at both peripheral and local levels (Karagiannis et al., 2015). Elevated mRNA expression of IL‐4, IL‐10 and IFN‐γ, cytokines known to drive and maintain CSR in favour of IgG_4_ (Ishizaka et al., 1990; King & Nutman, 1993; Satoguina et al., 2005), could predict higher IgG_4_ levels in both primary and metastatic CM when compared to healthy skin samples (Karagiannis et al., 2013). This CSR likely occurs in situ, as evidence in both melanoma and healthy skin shows antibodies with identical V(D)J sequences belonging to different subclasses occurs in parallel with the expression of AID (Saul et al., 2016). Finally, a subset of IgG_4_^+^ B‐cells (IgG_4_^+^CD49b^+^CD73^+^) that express pro‐angiogenic cytokines including VEGF, CYR61, ADM, FGF2, PDGFA and MDK has been identified; this subset is increased in the circulation of melanoma patients and could suggest a potential role for IgG_4_ in tumour angiogenesis (van de Veen et al., 2020).

There has only been one study that specifically examined the functional effect of increased IgG_4_ in melanoma. Antibodies with the same binding specificity to melanoma‐associated antigen CSPG4 (also known as MCSP) but differing in the Fc domains (IgG_1_ or IgG_4_) were tested for effector function; IgG_1_ mediated significantly higher levels of antibody‐dependent cell‐mediated phagocytosis (ADCP) of melanoma by monocytes than IgG_4_ (Karagiannis et al., 2013). Additionally, the degree of tumour death seen by IgG_1_ was lowered when IgG_4_ antibodies were concurrently present, implying IgG_4_ is not only unable to trigger ADCP, but its presence may also dampen IgG_1_‐mediated ADCP. Furthermore, the presence of IgG_4_ of irrelevant specificity can also decrease the IgG_1_‐mediated ADCP showing that competition for antigen binding may not be the only mechanism by which IgG_4_ modulation of immune response occurs (Karagiannis et al., 2013).

## 20 FUTURE RESEARCH DIRECTIONS AND CONCLUSIONS

As we have made clear throughout, before we can reach an understanding of the role of B‐cell subtypes, TLS and Ig subclasses in melanoma, the field at large needs to reach consensus on the markers used to identify them. It is currently difficult to reach conclusions on the roles of each of these factors when their definition differs between studies. Urgently, comprehensive guidelines should be produced, such as those put forward for TLS (Dieu‐Nosjean et al., 2014) or in response to the call for consistent classification of B‐cells (Sanz et al., 2019). There are a wide variety of areas in humoral immunity in melanoma that need further research. These include mechanisms controlling B‐cell subtype infiltration, better understanding of the epitopes and protein targets of the antibody response, the isotype and subclass of the Ig produced, the role of B‐cell epitope spreading, the role of B_regs_ in melanoma, and whether an immune‐suppressive TLS occurs after long‐term exposure to antigen as hypothesised in Figure 1a. Further, studies of B‐cells in melanomas have largely focussed on CM, so there are little data on the role of the humoral system in UM, ALM or MM. There are also still fundamental aspects of B‐cell biology coming to light, which might impact our understanding of the humoral response to melanoma. For example, challenging dogma, a subset of B‐cells have now been shown to have multiple BCR present and a single B‐cell can express multiple Ig classes, each showing unique V_H_DJ_H_ recombination patterns (Shi et al., 2019). As techniques such as scRNA‐seq technologies improve, in‐depth characterisation of B‐cell infiltration to melanomas will be revealed. Functional information on B‐cell subpopulations identified by scRNAseq can be inferred by the comparison of transcriptome profiles (Stewart et al., 2021). scRNA‐seq also allows the sequencing of paired heavy and light chains from individual B‐cells, which is more appropriate since both the heavy and light chain of an antibody contributes to the antigen‐binding specificity (Goldstein et al., 2019); however, the number of cells that can be sequenced with this approach is still limited. A study similar to that carried out in non‐small‐cell lung cancer (NSCLC) should be performed in melanoma, where scRNA‐seq was used to identify NSCLC‐infiltrating B‐cells at high resolution (Chen et al., 2020). Additional mechanistic studies following scRNA‐seq are also required. For example, in NSCLC, Chen et al. also revealed novel functions of tumour‐infiltrating B‐cells, including the inhibition of tumour growth in the presence of IgG^hi^ B‐cells in early stages of disease but the promotion of tumour growth at later stages of disease (Chen et al., 2020), which could indicate a switch in IgG subtype or IgG function. Further, they revealed a mechanism by which pathological antibodies could be transported into tumour cells via AP2 complexes and then degrade intracellular targets (Chen et al., 2020), suggesting a way that intracellularly targeted antibodies could play a role in tumour biology. This kind of in‐depth and mechanistic study is currently lacking in melanoma.

Antibodies are now a mainstay cancer treatment, specifically targeting tumour (e.g. anti‐EGFR and HER2) or immune cells (e.g. anti‐CTLA4, PD‐1 and PD‐L1) (Bhandaru & Rotte, 2019). These antibodies have been derived by identifying attractive targets and working towards antibody specificity. The emerging data from investigations of B‐cells and antibodies in melanoma suggest a future direction of research might be to work ‘forwards’ to identify existing antibody/antigen targets in patients to identify new personalised treatments to enhance these anti‐tumour responses. The isotype and subclass of identified Ig will be important to take into consideration, and the potential to modulate the Ig subtype away from an immune‐suppressive one requires more investigation.

After bringing together the existing literature on the humoral response, we propose some guidance to further our understanding of the roles of B‐cells and antibodies in melanoma. Firstly, the single‐pan B‐cell markers (CD19 or CD20) should not be used when examining associations between prognosis or response to treatment and the humoral response; it is vital that the function of the B‐cell response is taken into consideration. Secondly, the field as a whole must reach a better consensus of markers to identify B‐cell subtypes (e.g. such as proposed by (Sanz et al., 2019)) and the associated TLS (e.g. as proposed by (Hendry et al., 2017a, 2017b)). There is also the exciting potential to identify consistent subtype markers thanks to new technologies such as scRNA‐seq. Thirdly, more functional studies are required, to better understand the intricacies of the anti‐tumour or immunosuppressive humoral responses in melanomas. Finally, with a better understanding of the markers required to identify different functionally relevant aspects of the humoral response, we will be able to fully exploit new spatial‐omics technologies, which will provide vital context within the native spatial context of the tumour (Lewis et al., 2021).

In summary, despite the shortcomings we have outlined here, there are clearly important roles for B‐cells and antibodies in melanoma, including active and suppressive immune responses that influence the prognosis and progression of disease and the response to treatment with ICB. This is a field ready for significant advances in our understanding to be made, and with consistent and careful study design, advancements such as those seen in the T‐cell field will be possible.

## Supporting information

Fig S1

Table S1

Table S2

Supplementary Material

## Data Availability

Data sharing not applicable to this article as no datasets were generated or analysed during the current study.
