# Supplementary material for: A B‐cell or a key player? The different roles of B‐cells and antibodies in melanoma
Source: Pigment Cell Melanoma Res. 2022 Mar 4;35(3):303–19. doi: 10.1111/pcmr.13031 (PMC9314792; doi:10.1111/pcmr.13031)
Supplement: Supplementary file 1 — Fig S1 [file PCMR-35-303-s001.pdf]

A B CELL RECEPTOR (BCR) AND ANTIBODY ISOTYPES

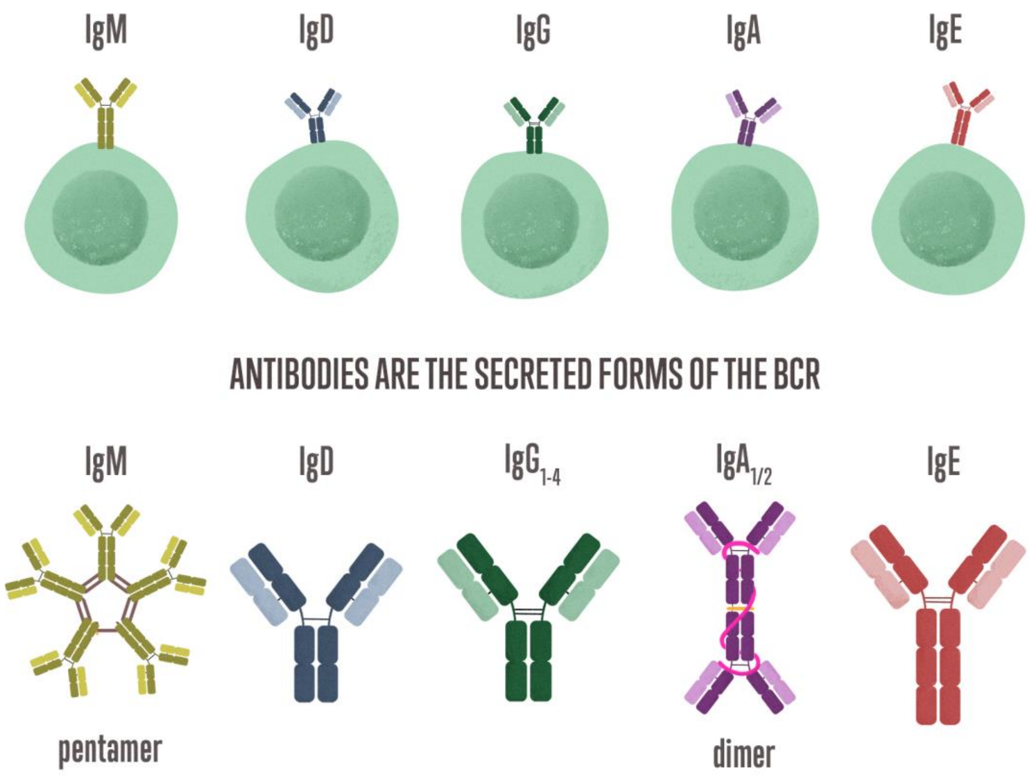

B ANTIBODY STRUCTURE

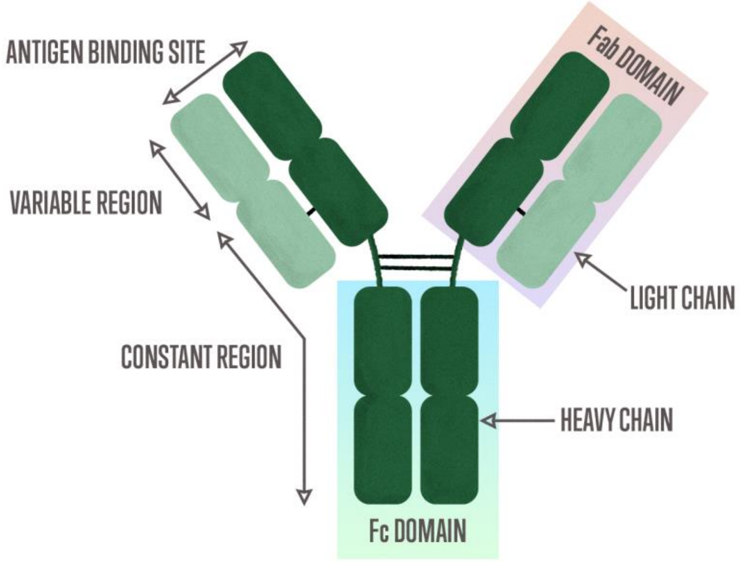

C CLASS SWITCH RECOMBINATION

only the constant region of the heavy chain is changed

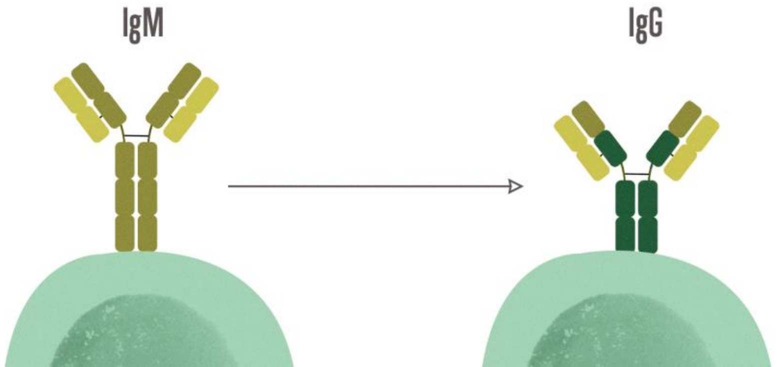

D ANTIBODY RECEPTOR e.g. FcγR (receptor for IgG)

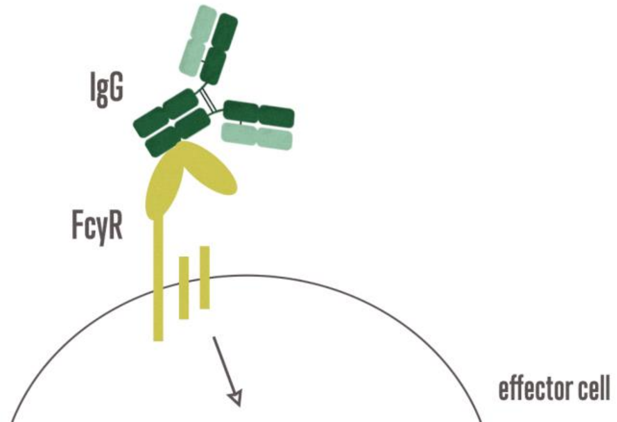

Supplementary Figure 1: Isotypes of antibodies produced by B-cells

(A) IgM is exclusively expressed by immature B-cells. IgD starts to be expressed when B-cells exit the bone marrow and when a B-cell reaches its mature state, it co-expresses IgM and IgD. IgM have the lowest antigen affinity and are frequently found as pentamers. Following somatic hypermutation and class switching, IgA, IgE or IgG can be produced. IgG is the most abundant immunoglobulin present in human serum and is produced in response to many different types of pathogen. (B) The Fc domain consists of the heavy chain, which changes during class switch recombination (C), meaning the antibody can interact with different effector cells via cell surface receptors called Fc receptors (e.g. (D), the FcγR binds IgG). The Fab domain, remains constant during class switch recombination (C), meaning specificity to antigen is retained. The Fab and Fc domains are linked by disulphide bonds.
