# Supplementary material for: A B‐cell or a key player? The different roles of B‐cells and antibodies in melanoma
Source: Pigment Cell Melanoma Res. 2022 Mar 4;35(3):303–19. doi: 10.1111/pcmr.13031 (PMC9314792; doi:10.1111/pcmr.13031)
Supplement: Supplementary file 2 — Table S1 [file PCMR-35-303-s004.docx]

**Supplementary Table 1: Identification of B-cell subtypes in non-melanoma solid tumours.**

The markers used to identify user-defined B-cell subtypes is detailed, along with the method used for detection and the tumours examined by the studies referenced.

| **Cell (subtype)** | **Method Used** | **Cancer Types Included** | **Markers used** | **Reference** |
| --- | --- | --- | --- | --- |
| B cell | Flow Cytometry, Immunohistochemistry | Lung, colorectal, breast, ductal breast, NSCLC, ovarian, high grade serous ovarian, SCC, HCC, HNSCC, oesophageal, pancreatic adenocarcinoma, gastric adenocarcinoma, OSCC, biliary, prostate, bladder, mesothelioma, large cell lung carcinoma, | CD20^+^ | [1-63] |
| B cell | Immunohistochemistry | Breast, HCC | CD79A^+^ | [16, 64, 65] |
| B cells | Immunohistochemistry | HNSCC, NSCLC | CD79^+^ | [18, 25] |
| B cells | Flow Cytometry, Immunohistochemistry | Ovarian, gastric | CD19^+^ | [66-69] |
| B-cells | Flow Cytometry | Colorectal | CD19^+^CD21^+^ | [70] |
| B cells | Flow Cytometry | HCC | CD19^+^CD24^-^CD38^+^ | [21] |
| Conventional B cells | Flow Cytometry | Pancreatic | CD19^+^CD24^lo^CD38^–^ | [71] |
| Conventional peripheral B cells | Flow Cytometry | HCC | CD5^+^CD24^hi^CD27^+^CD38^hi^ | [65] |
| Naïve B cells | Flow Cytometry | Breast | CD24^lo^CD27^−^ | [72] |
| Naïve B cells | Flow Cytometry | NSCLC | CD79A^+^CD27^−^IgD^+^ | [73] |
| Naïve B cells | Flow Cytometry | Ovarian | CD20^+^IgD^+^IgM^+^IgG^-^ | [10] |
| Naïve B cells | Flow Cytometry | HCC | IgD^+^IgM^+^IgG^-^ | [42] |
| Naïve B cells | Flow Cytometry | Ovarian | CD20^+^IgD^+^CD38^-/lo^ | [10] |
| Naïve B cells | Flow Cytometry | NCSLC | CD19^+^IgM^+^IgD^+^CD38^+/-^CD27^-^ | [74] |
| Naïve B cells | Flow Cytometry | OSCC | CD19^+^IgD^+^CD38^-^ | [26] |
| Naïve B cells | Flow Cytometry | Ovarian | IgD^+^IgG^-^CD38^-^ | [30] |
| Naïve B cells | Flow Cytometry | Ovarian | CD19^+^CD20^+^CD27^-^CD95^-^CD138^-^ | [30] |
| Naïve B cells | Flow Cytometry | HCC | CD45RA^+^BTLA^+^HLA^-^DR^+^IgD^+^IgM^+^IgG^-^ | [65] |
| Naïve B cells | Flow Cytometry | HNSCC | CD45^+^ CD19^+^CD20^+^IgD^+^CD27^-^ | [75] |
| Naïve B cells | Flow Cytometry | Ovarian | IgD^+^IgM^+^CD27^−^ | [76] |
| Naïve B cells | Flow Cytometry | High grade serous ovarian | CD27^-^IgM^+^ | [63] |
| Bm1 - naïve | Flow Cytometry | NSCLC | IgD^+^CD38^-^CD27^-^CD23^-^ | [5] |
| Bm2 - naïve | Flow Cytometry | NSCLC | IgD^+^CD38^-^CD27^-^CD23^+^ | [5] |
| Virgin naïve B cells | Flow Cytometry | Colorectal | CD19^+^CD20^+^IgD^+^CD27^-^CD38^-^ | [11] |
| Naïve-like B cell | Flow Cytometry, Immunohistochemistry | NSCLC | CD79A^+^CD20^+^ | [77] |
| Naïve IgD cells | Immunohistochemistry | Breast, NSCLC | IgD^+^ | [4, 5] |
| Resting naïve | Flow Cytometry | Breast | CD19^+^CD45^+^IgD^+^CD38^hi^ | [4] |
| Virgin activated B cell | Flow Cytometry | Colorectal | CD19^+^CD20^+^IgD^+^CD27^+^CD38^-^ | [11] |
| Activated naïve | Flow Cytometry | Breast | CD19^+^CD45^+^IgD^+^CD38^int^ | [4] |
| Activated B cell | Flow Cytometry | Ovarian | CD20^+^IgD^-^IgM^-^IgG^+^ | [10] |
| Activated B cells | Flow Cytometry | Colorectal | CD19^+^CD20^+^CD86^+^ | [11] |
| Activated B cells | Immunohistochemistry | HCC | CD20^+^CD27^+^ | [21] |
| Activated B cells | Flow Cytometry | HNSCC | CD45^+^CD19^+^CD20^+^CD86^+^ | [75] |
| AID+ B cells | Immunohistochemistry | Ovarian | CD20^+^AID^+^ | [30] |
| IgM+ memory B cells | Immunohistochemistry | Breast | IgM^+^ | [4] |
| IgM+ memory B cells | Flow Cytometry | High grade serous ovarian | CD27^+^IgM^+^ | [63] |
| Unswitched memory B cells | Flow Cytometry | Ovarian | IgD^−^IgM^+^CD27^+^ | [76] |
| Class switched memory B cells | Flow Cytometry | High grade serous ovarian | CD27^+^IgM^-^ | [63] |
| Class switched memory B cells | Flow Cytometry | Ovarian | IgD^−^IgM^−^CD27^+^ | [76] |
| Active/memory | Flow Cytometry | Breast, | CD24^hi^CD27^+^ | [72] |
| Early memory/germinal centre B cell | Flow Cytometry | NSCLC | CD79A^+^CD27^+^IgD^+^ | [73] |
| Early memory | Flow Cytometry | Breast | CD19^+^CD45^+^IgD^-^CD38^int^ | [4] |
| Bm5 early - memory | Flow Cytometry | NSCLC | IgD^-^CD23^-^CD27^+^CD38^+^ | [5] |
| Memory B-cell | Flow Cytometry | Ovarian | CD20^+^IgD^-^CD38^-/lo^ | [10] |
| Memory B cells | Flow Cytometry | Colorectal | CD19^+^CD20^+^IgD^-^CD27^+^ | [11] |
| Memory B cells | Flow Cytometry | OSCC | CD19^+^IgD^-^CD38^-^ | [26] |
| Memory B cells | Flow Cytometry | Ovarian | CD19^+^CD20^+^CD27^+^CD95^+^CD138^-^ | [30] |
| Memory B cells | Flow Cytometry | Ovarian | IgD^-^IgG^+^CD38^-^ | [30] |
| Memory B cells | Flow Cytometry | HCC | CD45RO^+^IgD^-^IgM^-^IgG^+^ | [65] |
| Memory B cells | Flow Cytometry | HNSCC | CD45^+^CD19^+^CD20^+^IgD^-^CD27^+^ | [75] |
| Atypical memory B cells | Flow Cytometry | HCC | IgD^-^IgG^+^CD27^-^CD38^-^ | [42] |
| Atypical memory B cells | Flow Cytometry | High grade serous ovarian | CD27^-^IgM^-^ | [63] |
| Bm5 late - memory | Flow Cytometry | NSCLC | IgD^-^CD23^-^CD27^+^CD38^-^ | [5] |
| "Double-negative memory B cell" | Flow Cytometry | NSCLC | CD79A^+^CD27^−^IgD^−^ | [73] |
| Pre-germinal centre | Flow Cytometry | Breast | CD19^+^CD45^+^IgD^+^CD38^lo^ | [4] |
| Pre-germinal centre | Flow Cytometry | OSCC | CD19^+^IgD^+^CD38^+^ | [26] |
| Proliferating germinal centre B cells | Immunohistochemistry | NSCLC | CD20^+^Ki67^+^ | [60] |
| Germinal centre | Flow Cytometry | Breast | CD19^+^CD45^+^IgD^-^CD38^hi^ | [4] |
| Germinal centre | Flow Cytometry | Ovarian | CD20^+^IgD^-^CD38^+^ | [10] |
| Germinal centre | Flow Cytometry | OSCC | CD19^+^IgD^-^CD38^+^ | [26] |
| Germinal centre | Immunohistochemistry | Breast | CD20^+^IgD^-^PD1^+^ CD35^+^IgM^+^Ki67^+^CD138^+^ | [4] |
| Germinal centre | Immunohistochemistry | Ovarian | CD20^+^CD3^+^Bcl6^+^ and AID^+^CD20^+^ | [30] |
| Germinal centre | Immunohistochemistry | Gastric | CD20^+^CD3^+^Ki67^+^DC-Lamp^+^ | [24] |
| Germinal centre | Immunohistochemistry | Pancreatic | CD20^+^Ki67^+^Bcl6^+^ | [17] |
| Germinal centre | Immunohistochemistry | NSCLC | CD23^+^AID^+^Ki67^+^Bcl6^+^Bcl2^-^ | [5] |
| Germinal centre | Immunohistochemistry | Gastric | Bcl6+ | [39] |
| Germinal centre | Immunohistochemistry | HNSCC | CD21^+^CD57^+^ | [46] |
| Germinal centre | Immunohistochemistry | NSCLC | Bcl6^+^CD21^+^ | [78] |
| Germinal centre | Immunohistochemistry | Colorectal | CD20^+^Ki67^+^ | [11] |
| Germinal centre | Immunohistochemistry | High grade serous ovarian | CD45RO^+^Ki67^+^ and CD20^+^FDC^+^ (marker not defined) | [63] |
| Bm3 - Germinal centre | Flow Cytometry | NSCLC | IgD^-^CD38^+^CD23^-^CD27^-^CD77^+^ | [5] |
| Bm4 - Germinal centre | Flow Cytometry | NSCLC | IgD^-^CD38^+^CD23^-^CD27^-^CD77^-^ | [5] |
| Germinal centre memory B cell | Flow Cytometry | NSCLC | CD19^+^IgM^-^IgD^-^CD27^+^CD38^+/-^ | [74] |
| Affinity-matured B cell | Flow Cytometry | NSCLC | CD79A^+^CD27^+^IgD^−^ | [73] |
| Active mature | Flow Cytometry | HCC | IgD^-^IgM^-/lo^IgG^+^ | [42] |
| Atypical memory B cells | Flow Cytometry | Ovarian | CD20^+^IgG^+^CD27^-^ | [10] |
| Late memory | Flow Cytometry | Breast | CD19^+^CD45^+^IgD^-^CD38^lo^ | [4] |
| Plasmablast | Flow Cytometry | Ovarian | CD20^-^CD38^+^CD138^-^CD3^-^CD56^-^ | [10] |
| Plasmablast | Flow Cytometry | Pancreatic | CD19^+^CD24^hi^CD27^int^ | [71] |
| Plasmablast | Flow Cytometry | HNSCC | CD45^+^CD19^+^CD20^-^CD27^+^CD38^hi^ | [75] |
| Plasmablasts and plasma cells | Flow Cytometry | Breast | CD27^hi^CD38^hi^ | [4] |
| Plasma cell/Activated T-cell | Immunohistochemistry | Lung | Mum1 | [1] |
| Plasma cells | Immunohistochemistry | Colorectal, breast, NSCLC, oesophageal and gastric adenocarcinoma, SCC, bladder | CD138^+^ | [2, 4, 5, 7, 19, 29, 34, 36, 37, 47, 49, 50, 79] |
| Plasma cells | Immunohistochemistry | Ovarian | CD138^+^IGKC^+^ | [8] |
| Plasma cells | Immunohistochemistry | Colorectal, oesophageal and gastric adenocarcinoma | IGKC^+^ | [2, 7, 11, 19] |
| Plasma cells | Flow Cytometry | Breast | CD38^hi^CD138^+^ | [4] |
| Plasma cells | Flow Cytometry | Ovarian | CD20^-^CD38^+^CD138^+^ | [10] |
| Plasma cells | Flow Cytometry | NSCLC | IgD^-^CD38^++^CD138^-^ | [5] |
| Plasma cells | Flow Cytometry | Colorectal, NSCLC, High grade serous ovarian cancer | CD19^+^CD20^-^CD27^+^CD38^hi^ | [11, 63, 74] |
| Plasma cells | Immunohistochemistry | HCC | CD27^+^CD38^+^CD138^+^ | [21] |
| Plasma cells | Flow Cytometry | HCC | CD19^-^CD27^+^CD38^+^CD138^+^ | [21] |
| Plasma cells | Flow Cytometry | Ovarian | IgD^-^IgG^+^CD38^+^ | [30] |
| Plasma cells | Flow Cytometry | Ovarian | CD19^+^CD20^-^CD27^+^CD95^+^CD138^lo^ | [30] |
| Plasma cells | Flow Cytometry | OSCC | CD19^+^IgD^-^CD38^++^ | [26] |
| Plasma cells | Immunohistochemistry | Ovarian | CD38^+^CD138^+^CD79a^+^ | [30] |
| Plasma cells | Immunohistochemistry | Lung adenocarcinoma | CD79^+^p63^+^ | [31] |
| Plasma cells | Flow Cytometry | HNSCC | CD45^+^CD19^+^CD20^-^CD27^+^CD38^hi^CD138^hi^ | [75] |
| Plasma cells | Immunohistochemistry | Breast | CD38^+^ | [41] |
| Mature plasma cells | Flow Cytometry | NSCLC | IgD^-^CD38^++^CD138^+^ | [5] |
| Plasma-like B cell | Flow Cytometry, Immunohistochemistry | NSCLC | CD79A^+^CD20^-^ | [77] |
| IgG Kappa^+^CD38^+^ plasma cells | Immunohistochemistry | Breast | IGKC^+^CD38^+^ | [41] |
| IgA^+^CD38^+^ plasma cells | Immunohistochemistry | Breast | IgA^+^CD38^+^ | [41] |
| CD138^+^CD38^+^ plasma cells | Immunohistochemistry | Breast | CD138^+^CD38^+^ | [41] |
| PD-1^+^CD38^+^ plasma cells | Immunohistochemistry | Breast | PD-1^+^CD38^+^ | [41] |
| Immature regulatory B cell | Flow Cytometry | Pancreatic | CD19^+^CD24^hi^CD38^hi^ | [71] |
| Transitional B cell, regulatory | Flow Cytometry | Colorectal | CD19^+^CD20^+^CD24^hi^CD38^hi^ | [11] |
| Regulatory B cell subtype | Flow Cytometry | Colorectal | CD19^+^CD20^+^CD24^hi^CD27^+^ | [11] |
| Regulatory B cells | Flow Cytometry | HCC | CD19^+^CD24^+^CD38^+^ | [21] |
| Regulatory B cells | Flow Cytometry | OSCC | CD5^+^CD24^hi^ | [26] |
| Regulatory B cells (or “IL-10 producing B cells” [68, 80]) | Flow Cytometry, Immunohistochemistry | Gastric, breast, tongue SCC | CD19^+^IL10^+^ | [68, 69, 80-82] |
| Regulatory B cells | Flow Cytometry | Gastric | CD24^+^ | [39] |
| Regulatory B cells | Immunohistochemistry | Bladder cancer | CD20^+^IL10^+^ | [50] |
| Regulatory B cells | Immunohistochemistry | Breast | CD19^+^CD25^+^ | [82] |
| Regulatory B cell | Flow Cytometry | HNSCC | CD45^+^CD19^+^CD25^hi^ | [75] |
| Regulatory B cell | Flow Cytometry | HNSCC, gastric | CD45^+^CD19^+^CD24^hi^CD38^hi^ | [68, 75] |
| Regulatory B cells | Flow Cytometry | HCC | CD24^hi^CD38^hi^ | [65] |
| Br1 Regulatory B cells | Flow Cytometry | Pancreatic | CD19^+^CD25^hi^CD71^+^ | [71] |
| Br10 Regulatory B cells | Flow Cytometry | Pancreatic | CD19^+^CD24^hi^CD27^+^ (IL10 confirmed by qPCR) | [71] |
| B10 B cells | Flow Cytometry | HCC | CD5^+^CD19^+^IL10^+^ | [83] |
| B10 B cells | Flow Cytometry | OSCC | CD19^+^CD24^hi^CD27^+^ (CD19^+^IL10^+^ cells were then assessed in patients vs. controls) | [84] |
| IL-10 B cell | Flow Cytometry | Ovarian | CD19^+^CD20^+^IL-10^+^ | [76] |
| Activated TIL-B cell | Flow Cytometry | NSCLC | CD19^+^CD20^+^CD21^+^CD27^+^CD69^+^ | [85] |
| Exhausted TIL-B cell | Flow Cytometry | NSCLC | CD19^+^CD20^+^CD21^-^CD27^-^CD69^+^ | [85] |
| PD-1 high B cells | Flow Cytometry | HCC | CD5^hi^CD24^−/+^CD27^hi/+^CD38^dim^ | [65] |
| IL-35 producing B cells | Flow Cytometry | Gastric | CD19^+^EBI3^+^p35^+^ | [80] |
| IgM+ IgD- B cells | Flow Cytometry | NSCLC | CD19^+^IgM^+^IgD^-^ | [74] |
| Antigen presenting cells | Flow Cytometry | HNSCC | CD45^+^CD19^+^CD20^+^CD21^-^CD86^+^ | [75] |

NSCLC: Non-small cell lung cancer; SCC: squamous cell carcinomas; HCC: hepatocellular carcinoma; OSCC: Oropharyngeal squamous cell carcinoma; HNSCC: head and neck squamous cell carcinoma; IGKC: Immunoglobulin kappa C

**References**

1. Banat, G.A., et al., *Immune and Inflammatory Cell Composition of Human Lung Cancer Stroma.* PLoS One, 2015. **10**(9): p. e0139073.

2. Berntsson, J., et al., *Prognostic impact of tumour-infiltrating B cells and plasma cells in colorectal cancer.* Int J Cancer, 2016. **139**(5): p. 1129-39.

3. Edin, S., et al., *The Prognostic Importance of CD20(+) B lymphocytes in Colorectal Cancer and the Relation to Other Immune Cell subsets.* Sci Rep, 2019. **9**(1): p. 19997.

4. Garaud, S., et al., *Tumor infiltrating B-cells signal functional humoral immune responses in breast cancer.* JCI Insight, 2019. **5**.

5. Germain, C., et al., *Presence of B cells in tertiary lymphoid structures is associated with a protective immunity in patients with lung cancer.* Am J Respir Crit Care Med, 2014. **189**(7): p. 832-44.

6. Gu, Y., et al., *Tumor-educated B cells selectively promote breast cancer lymph node metastasis by HSPA4-targeting IgG.* Nat Med, 2019. **25**(2): p. 312-322.

7. Lohr, M., et al., *The prognostic relevance of tumour-infiltrating plasma cells and immunoglobulin kappa C indicates an important role of the humoral immune response in non-small cell lung cancer.* Cancer Lett, 2013. **333**(2): p. 222-8.

8. Lundgren, S., et al., *Prognostic impact of tumour-associated B cells and plasma cells in epithelial ovarian cancer.* J Ovarian Res, 2016. **9**: p. 21.

9. Mahmoud, S.M., et al., *The prognostic significance of B lymphocytes in invasive carcinoma of the breast.* Breast Cancer Res Treat, 2012. **132**(2): p. 545-53.

10. Nielsen, J.S., et al., *CD20+ tumor-infiltrating lymphocytes have an atypical CD27- memory phenotype and together with CD8+ T cells promote favorable prognosis in ovarian cancer.* Clin Cancer Res, 2012. **18**(12): p. 3281-92.

11. Shimabukuro-Vornhagen, A., et al., *Characterization of tumor-associated B-cell subsets in patients with colorectal cancer.* Oncotarget, 2014. **5**(13): p. 4651-64.

12. Del Mar Valenzuela-Membrives, M., et al., *Progressive changes in composition of lymphocytes in lung tissues from patients with non-small-cell lung cancer.* Oncotarget, 2016. **7**(44): p. 71608-71619.

13. Affara, N.I., et al., *B cells regulate macrophage phenotype and response to chemotherapy in squamous carcinomas.* Cancer Cell, 2014. **25**(6): p. 809-821.

14. Al-Shibli, K.I., et al., *Prognostic effect of epithelial and stromal lymphocyte infiltration in non-small cell lung cancer.* Clin Cancer Res, 2008. **14**(16): p. 5220-7.

15. Baeten, C.I., et al., *Proliferating endothelial cells and leukocyte infiltration as prognostic markers in colorectal cancer.* Clin Gastroenterol Hepatol, 2006. **4**(11): p. 1351-7.

16. Brunner, S.M., et al., *Tumor-infiltrating B cells producing antitumor active immunoglobulins in resected HCC prolong patient survival.* Oncotarget, 2017. **8**(41): p. 71002-71011.

17. Castino, G.F., et al., *Spatial distribution of B cells predicts prognosis in human pancreatic adenocarcinoma.* Oncoimmunology, 2016. **5**(4): p. e1085147.

18. Distel, L.V., et al., *Tumour infiltrating lymphocytes in squamous cell carcinoma of the oro- and hypopharynx: prognostic impact may depend on type of treatment and stage of disease.* Oral Oncol, 2009. **45**(10): p. e167-74.

19. Fristedt, R., et al., *Prognostic impact of tumour-associated B cells and plasma cells in oesophageal and gastric adenocarcinoma.* J Gastrointest Oncol, 2016. **7**(6): p. 848-859.

20. Gao, Q., et al., *Infiltrating memory/senescent T cell ratio predicts extrahepatic metastasis of hepatocellular carcinoma.* Ann Surg Oncol, 2012. **19**(2): p. 455-66.

21. Garnelo, M., et al., *Interaction between tumour-infiltrating B cells and T cells controls the progression of hepatocellular carcinoma.* Gut, 2017. **66**(2): p. 342-351.

22. Goeppert, B., et al., *Prognostic impact of tumour-infiltrating immune cells on biliary tract cancer.* Br J Cancer, 2013. **109**(10): p. 2665-74.

23. Haas, M., et al., *Stromal regulatory T-cells are associated with a favourable prognosis in gastric cancer of the cardia.* BMC Gastroenterol, 2009. **9**: p. 65.

24. Hennequin, A., et al., *Tumor infiltration by Tbet+ effector T cells and CD20+ B cells is associated with survival in gastric cancer patients.* Oncoimmunology, 2016. **5**(2): p. e1054598.

25. Hernandez-Prieto, S., et al., *A 50-gene signature is a novel scoring system for tumor-infiltrating immune cells with strong correlation with clinical outcome of stage I/II non-small cell lung cancer.* Clin Transl Oncol, 2015. **17**(4): p. 330-8.

26. Hladikova, K., et al., *Tumor-infiltrating B cells affect the progression of oropharyngeal squamous cell carcinoma via cell-to-cell interactions with CD8(+) T cells.* J Immunother Cancer, 2019. **7**(1): p. 261.

27. Kasajima, A., et al., *Down-regulation of the antigen processing machinery is linked to a loss of inflammatory response in colorectal cancer.* Hum Pathol, 2010. **41**(12): p. 1758-69.

28. Kinoshita, T., et al., *Prognostic value of tumor-infiltrating lymphocytes differs depending on histological type and smoking habit in completely resected non-small-cell lung cancer.* Ann Oncol, 2016. **27**(11): p. 2117-2123.

29. Knief, J., et al., *High Density of Tumor-infiltrating B-Lymphocytes and Plasma Cells Signifies Prolonged Overall Survival in Adenocarcinoma of the Esophagogastric Junction.* Anticancer Res, 2016. **36**(10): p. 5339-5345.

30. Kroeger, D.R., K. Milne, and B.H. Nelson, *Tumor-Infiltrating Plasma Cells Are Associated with Tertiary Lymphoid Structures, Cytolytic T-Cell Responses, and Superior Prognosis in Ovarian Cancer.* Clin Cancer Res, 2016. **22**(12): p. 3005-15.

31. Kurebayashi, Y., et al., *Comprehensive Immune Profiling of Lung Adenocarcinomas Reveals Four Immunosubtypes with Plasma Cell Subtype a Negative Indicator.* Cancer Immunol Res, 2016. **4**(3): p. 234-47.

32. Lee, H.E., et al., *Prognostic implications of type and density of tumour-infiltrating lymphocytes in gastric cancer.* Br J Cancer, 2008. **99**(10): p. 1704-11.

33. Liang, J., et al., *Expression pattern of tumour-associated antigens in hepatocellular carcinoma: association with immune infiltration and disease progression.* Br J Cancer, 2013. **109**(4): p. 1031-9.

34. Meshcheryakova, A., et al., *B cells and ectopic follicular structures: novel players in anti-tumor programming with prognostic power for patients with metastatic colorectal cancer.* PLoS One, 2014. **9**(6): p. e99008.

35. Mlecnik, B., et al., *Comprehensive Intrametastatic Immune Quantification and Major Impact of Immunoscore on Survival.* J Natl Cancer Inst, 2018. **110**(1).

36. Mohammed, Z.M., et al., *The relationship between lymphocyte subsets and clinico-pathological determinants of survival in patients with primary operable invasive ductal breast cancer.* Br J Cancer, 2013. **109**(6): p. 1676-84.

37. Nakajima, M., et al., *Tumor immune systems in esophageal cancer with special reference to heat-shock protein 70 and humoral immunity.* Anticancer Res, 2009. **29**(5): p. 1595-606.

38. Ness, N., et al., *Infiltration of CD8+ lymphocytes is an independent prognostic factor of biochemical failure-free survival in prostate cancer.* Prostate, 2014. **74**(14): p. 1452-61.

39. Sakimura, C., et al., *B cells in tertiary lymphoid structures are associated with favorable prognosis in gastric cancer.* J Surg Res, 2017. **215**: p. 74-82.

40. Santoiemma, P.P., et al., *Systematic evaluation of multiple immune markers reveals prognostic factors in ovarian cancer.* Gynecol Oncol, 2016. **143**(1): p. 120-127.

41. Seow, D.Y.B., et al., *Tertiary lymphoid structures and associated plasma cells play an important role in the biology of triple-negative breast cancers.* Breast Cancer Res Treat, 2020. **180**(2): p. 369-377.

42. Shi, J.Y., et al., *Margin-infiltrating CD20(+) B cells display an atypical memory phenotype and correlate with favorable prognosis in hepatocellular carcinoma.* Clin Cancer Res, 2013. **19**(21): p. 5994-6005.

43. Song, I.H., et al., *Predictive Value of Tertiary Lymphoid Structures Assessed by High Endothelial Venule Counts in the Neoadjuvant Setting of Triple-Negative Breast Cancer.* Cancer Res Treat, 2017. **49**(2): p. 399-407.

44. Sorbye, S.W., et al., *Prognostic impact of lymphocytes in soft tissue sarcomas.* PLoS One, 2011. **6**(1): p. e14611.

45. Tewari, N., et al., *The presence of tumour-associated lymphocytes confers a good prognosis in pancreatic ductal adenocarcinoma: an immunohistochemical study of tissue microarrays.* BMC Cancer, 2013. **13**: p. 436.

46. van Herpen, C.M., et al., *Intratumoral rhIL-12 administration in head and neck squamous cell carcinoma patients induces B cell activation.* Int J Cancer, 2008. **123**(10): p. 2354-61.

47. Vassallo, J., et al., *Pathologic and imunohistochemical characterization of tumoral inflammatory cell infiltrate in invasive penile squamous cell carcinomas: Fox-P3 expression is an independent predictor of recurrence.* Tumour Biol, 2015. **36**(4): p. 2509-16.

48. Wang, W.Q., et al., *Infiltrating immune cells and gene mutations in pancreatic ductal adenocarcinoma.* Br J Surg, 2016. **103**(9): p. 1189-99.

49. Yeong, J., et al., *High Densities of Tumor-Associated Plasma Cells Predict Improved Prognosis in Triple Negative Breast Cancer.* Front Immunol, 2018. **9**: p. 1209.

50. Zirakzadeh, A.A., et al., *Tumour-associated B cells in urothelial urinary bladder cancer.* Scand J Immunol, 2020. **91**(2): p. e12830.

51. Lu, L., et al., *IL-17A promotes migration and tumor killing capability of B cells in esophageal squamous cell carcinoma.* Oncotarget, 2016. **7**(16): p. 21853-64.

52. Pelletier, M.P., et al., *Prognostic markers in resectable non-small cell lung cancer: a multivariate analysis.* Can J Surg, 2001. **44**(3): p. 180-8.

53. Eerola, A.-K., Y. Soini, and P. Pääkkö, *Tumour infiltrating lymphocytes in relation to tumour angiogenesis, apoptosis and prognosis in patients with large cell lung carcinoma.* Lung Cancer, 1999. **26**(2): p. 73-83.

54. Schalper, K.A., et al., *Objective measurement and clinical significance of TILs in non-small cell lung cancer.* J Natl Cancer Inst, 2015. **107**(3).

55. Hald, S.M., et al., *CD4/CD8 co-expression shows independent prognostic impact in resected non-small cell lung cancer patients treated with adjuvant radiotherapy.* Lung Cancer, 2013. **80**(2): p. 209-15.

56. Suzuki, K., et al., *Clinical impact of immune microenvironment in stage I lung adenocarcinoma: tumor interleukin-12 receptor beta2 (IL-12Rbeta2), IL-7R, and stromal FoxP3/CD3 ratio are independent predictors of recurrence.* J Clin Oncol, 2013. **31**(4): p. 490-8.

57. Chee, S.J., et al., *Evaluating the effect of immune cells on the outcome of patients with mesothelioma.* Br J Cancer, 2017. **117**(9): p. 1341-1348.

58. Ujiie, H., et al., *The tumoral and stromal immune microenvironment in malignant pleural mesothelioma: A comprehensive analysis reveals prognostic immune markers.* Oncoimmunology, 2015. **4**(6): p. e1009285.

59. Hiraoka, N., et al., *Intratumoral tertiary lymphoid organ is a favourable prognosticator in patients with pancreatic cancer.* Br J Cancer, 2015. **112**(11): p. 1782-90.

60. Dieu-Nosjean, M.C., et al., *Long-term survival for patients with non-small-cell lung cancer with intratumoral lymphoid structures.* J Clin Oncol, 2008. **26**(27): p. 4410-7.

61. Brown, J.R., et al., *Multiplexed quantitative analysis of CD3, CD8, and CD20 predicts response to neoadjuvant chemotherapy in breast cancer.* Clin Cancer Res, 2014. **20**(23): p. 5995-6005.

62. Milne, K., et al., *Systematic analysis of immune infiltrates in high-grade serous ovarian cancer reveals CD20, FoxP3 and TIA-1 as positive prognostic factors.* PLoS One, 2009. **4**(7): p. e6412.

63. Montfort, A., et al., *A Strong B-cell Response Is Part of the Immune Landscape in Human High-Grade Serous Ovarian Metastases.* Clin Cancer Res, 2017. **23**(1): p. 250-262.

64. Romaniuk, A. and C.i.U.M. Lsmall u, *Immune microenvironment as a factor of breast cancer progression.* Diagn Pathol, 2015. **10**: p. 79.

65. Xiao, X., et al., *PD-1hi Identifies a Novel Regulatory B-cell Population in Human Hepatoma That Promotes Disease Progression.* Cancer Discov, 2016. **6**(5): p. 546-59.

66. Dong, H.P., et al., *NK- and B-Cell Infiltration Correlates With Worse Outcome in Metastatic Ovarian Carcinoma.* American Journal of Clinical Pathology, 2006. **125**(3): p. 451-458.

67. Yang, C., et al., *Prognostic significance of B-cells and pSTAT3 in patients with ovarian cancer.* PLoS One, 2013. **8**(1): p. e54029.

68. Wang, W.W., et al., *CD19+CD24hiCD38hiBregs involved in downregulate helper T cells and upregulate regulatory T cells in gastric cancer.* Oncotarget, 2015. **6**(32): p. 33486-99.

69. Zhou, X., et al., *CD19(+)IL-10(+) regulatory B cells affect survival of tongue squamous cell carcinoma patients and induce resting CD4(+) T cells to CD4(+)Foxp3(+) regulatory T cells.* Oral Oncol, 2016. **53**: p. 27-35.

70. Barbera-Guillem, E., et al., *B lymphocyte pathology in human colorectal cancer. Experimental and clinical therapeutic effects of partial B cell depletion.* Cancer Immunol Immunother, 2000. **48**(10): p. 541-9.

71. Mirlekar, B., et al., *B cell-Derived IL35 Drives STAT3-Dependent CD8(+) T-cell Exclusion in Pancreatic Cancer.* Cancer Immunol Res, 2020. **8**(3): p. 292-308.

72. Arabpour, M., et al., *Granzyme B production by activated B cells derived from breast cancer-draining lymph nodes.* Mol Immunol, 2019. **114**: p. 172-178.

73. Centuori, S.M., et al., *Double-negative (CD27(-)IgD(-)) B cells are expanded in NSCLC and inversely correlate with affinity-matured B cell populations.* J Transl Med, 2018. **16**(1): p. 30.

74. Stankovic, B., et al., *Immune Cell Composition in Human Non-small Cell Lung Cancer.* Front Immunol, 2018. **9**: p. 3101.

75. Lechner, A., et al., *Tumor-associated B cells and humoral immune response in head and neck squamous cell carcinoma.* Oncoimmunology, 2019. **8**(3): p. 1535293.

76. Wei, X., et al., *Regulatory B cells contribute to the impaired antitumor immunity in ovarian cancer patients.* Tumour Biol, 2016. **37**(5): p. 6581-8.

77. Chen, J., et al., *Single-cell transcriptome and antigen-immunoglobin analysis reveals the diversity of B cells in non-small cell lung cancer.* Genome Biol, 2020. **21**(1): p. 152.

78. Gottlin, E.B., et al., *The Association of Intratumoral Germinal Centers with early-stage non-small cell lung cancer.* J Thorac Oncol, 2011. **6**(10): p. 1687-90.

79. Al-Shibli, K., et al., *The prognostic value of intraepithelial and stromal CD3-, CD117- and CD138-positive cells in non-small cell lung carcinoma.* APMIS, 2010. **118**(5): p. 371-82.

80. Wang, K., J. Liu, and J. Li, *IL-35-producing B cells in gastric cancer patients.* Medicine (Baltimore), 2018. **97**(19): p. e0710.

81. Murakami, Y., et al., *Increased regulatory B cells are involved in immune evasion in patients with gastric cancer.* Sci Rep, 2019. **9**(1): p. 13083.

82. Ishigami, E., et al., *Coexistence of regulatory B cells and regulatory T cells in tumor-infiltrating lymphocyte aggregates is a prognostic factor in patients with breast cancer.* Breast Cancer, 2019. **26**(2): p. 180-189.

83. Qian, L., et al., *Clinical significance of regulatory B cells in the peripheral blood of patients with oesophageal cancer.* Cent Eur J Immunol, 2015. **40**(2): p. 263-5.

84. Mao, Y., et al., *Circulating exosomes from esophageal squamous cell carcinoma mediate the generation of B10 and PD-1(high) Breg cells.* Cancer Sci, 2019. **110**(9): p. 2700-2710.

85. Bruno, T.C., et al., *Antigen-Presenting Intratumoral B Cells Affect CD4(+) TIL Phenotypes in Non-Small Cell Lung Cancer Patients.* Cancer Immunol Res, 2017. **5**(10): p. 898-907.
