# Supplementary material for: A B‐cell or a key player? The different roles of B‐cells and antibodies in melanoma
Source: Pigment Cell Melanoma Res. 2022 Mar 4;35(3):303–19. doi: 10.1111/pcmr.13031 (PMC9314792; doi:10.1111/pcmr.13031)
Supplement: Supplementary file 3 — Table S2 [file PCMR-35-303-s003.docx]

**Supplementary Table 2: Antibody biomarkers described for detection of melanoma and/or disease progression in melanoma**

| **Citation** | **No. of Individuals tested** | **Method used** | **Antigen/Antibody tested** | **Results** |
| --- | --- | --- | --- | --- |
| (Stockert et al., 1998) | Melanoma patients  234 patients, including 127 with metastatic disease and tumours from 62 melanoma patients  Controls  70 healthy | ELISA | NY-ESO-1, MAGE-1, MAGE-3, SSX2, Melan-A, tyrosinase | 9.4% (12/127) metastatic melanoma patients had antibodies against NY-ESO-1. 8/15 NY-ESO positive tumours were in NY-ESO antibody positive patients.  Rarely antibodies were detected against MAGE1, MAGE3 and SSX2 in melanoma patients.  No antibody responses were found against Melan-A, tyrosinase, or carbonic anhydrase and no healthy controls had antibodies against any of these antigens. |
| (Huang et al., 1998) | Melanoma patients  23 melanoma patients pre and post treatment with polyvalent melanoma cell vaccine  Controls  23 healthy controls | ELISA and Western Blot | Tyrosinase, TRP1, TRP2, gp100 | Melanoma patients had autoantibody responses against the melanoma-associated antigens and the vaccine increased antibody response in some patients.  Only 1/23 controls had an antibody response to the antigens tested. |
| (Hodi et al., 2002) | Melanoma patients  8 melanoma patients with metastatic disease  Vaccinated with autologous GM-CSF-secreting tumour cells studied over a period of 200 days before and after vaccination. | ELISA | IgG antibodies against ATP6S1 | A vaccine-induced elevation of ATP6S1 IgG autoantibodies in 4/8 patients. In one case levels peaked prior to subcutaneous metastasis resection and then returned to baseline afterwards. |
| (Litvak et al., 2004) | Melanoma Patients  Primary melanomas 1.01 to 2.00 mm and negative regional lymph nodes were split by outcome:  Group 1: 50 patients who died of within 7 years  Group 2: 50 patients who were matched with group 1 but lived 10+ years without recurrence. | TA-90 antigen specific ELISA | TA-90 | A positive TA90-IgG immune complex test and absence of an anti-TA90 IgM response correlated with distant metastasis when melanoma is low risk or intermediate risk by standard prognostic factors. |
| (Gogas et al., 2006) | Melanoma patients  200 melanoma patients with stage IIB, IIC or III melanoma treated with high-dose adjuvant interferon alfa-2b | ELISA  Blood taken before interferon therapy, and after 1,3,6,9, and 12 months post-treatment | Antithyroid, antinuclear, anti-DNA, anticardiolipin autoantibodies | The presence of autoantibodies or clinical manifestations of autoimmunity during treatment was associated with significantly improved relapse-free survival and overall survival.  Median time to the detection of autoantibodies after treatment onset was 3 months. |
| (Nesterova et al., 2006) | Melanoma patients  90 melanoma patients, amongst many other cancers  Controls  163 healthy sera | ELISA | Anti-ECPKA antibodies | Patients with melanoma have anti-ECPKA antibodies, but this was not melanoma specific, as was found in many other cancer types. |
| (Bazhin et al., 2007) | Melanoma patients  72 melanoma patients  (Stage not specified)  Controls  72 healthy | Western blot | Autoantibodies against GAGE family proteins – (but only use GAGE-7b-tag?) | No healthy controls had autoantibodies against GAGE-7b-tag, but 4/72 (6%) of melanoma patients did. |
| (Zippelius et al., 2007) | Melanoma patients  Serum from 52 melanoma patients  Controls  15 non-melanoma cancer patients  13 vitiligo patients  15 healthy | ELISA using recombinant RAB38, 25 overlapping peptides of 18aa length derived from RAB38 and tetanus toxoid | Melanocyte differentiation antigen RAB38 | Anti-RAB38 antibodies were found exclusively in melanoma patients and not in other disease, in 12/52 patients (23%).  The general immune competence of the individuals was analysed via vaccine-induced responses against a recombinant tetanus toxoid protein. The frequency of tetanus-toxoid-specific antibody responses was not significantly different between cohorts. |
| (Vercambre-Darras et al., 2007) | Melanoma patients  10 long-term survivors of metastatic melanoma (3 had been treated with IFN-α)  10 recently diagnosed stage IV melanoma patients (2 had been treated with IFN-α) | Method not described | Antithyroid antibodies | Half of the long-term survivors had high frequency of antithyroid abs compared to only 1 of the more recently diagnosed patients.  4/5 patients with high autoantibodies were female. |
| (Shimbo et al., 2010) | Melanoma patients  55 melanoma patients (18 early stage I or II; 37 stage III or IV)  Controls  2 healthy volunteers | ELISA | Anti-BPAG1 autoantibodies  (found in the skin disease bullous pemphigoid and used for diagnosis) | Levels of anti-BPAG1 autoantibodies was higher in melanoma vs controls. |
| (Sabel et al., 2011, Liu et al., 2010) | Melanoma patients  79 patients | protein microarray | Glycoproteins: GRP75, GRP94, ASAH1, CTSD and LDHB | ASAH1, CTSD and LDHB were significantly negatively associated with nodal disease. Higher levels of autoantibodies against GRP94 was associated with a higher risk of nodal disease. |
| (Yuan et al., 2011) | Melanoma patients  144 advanced melanoma patients  Pre-treatment and after receiving Ipilimumab | ELISA | NY-ESO-1 | 17% of patients had pre-existing antibodies to NY-ESO-1.  Baseline seropositive patients had better outcome; those with additional CD8^+^ T-cell response has significantly better survival. |
| (Maire et al., 2013) | Melanoma patients  113 stage IV melanoma patients | Antithyroid and antinuclear autoantibody assays | Spontaneous autoantibodies: Antithyroid antibodies and  Antinuclear antibodies | Longer survival in the presence of antithyroid or antinuclear self-antibodies.  No correlation with gender. |
| (Zornig et al., 2015) | Melanoma patients  Total: 365 (97 Stage I, 87 Stage II, 92 Stage III and 89 Stage IV)  Controls  100 healthy, age and gender matched | Luminex bead‐based multiplex assay to detect patient specific IgG | Arrestin, CAMEL, CT47, Cyclin B1, GAGE7b, gp100, LAGE1a, MAGE‐A1, MAGE‐A3, MAGE‐A4, MAGE‐A9, MelanA, NY‐ESO‐1, OY‐TES‐1, p16, p53, Rab38, Recoverin, SpanXa1, SSX2, SSX4, Survivin, Tyrosinase, Muc1, Rhodopsin, cTAGE5a | 87% of the healthy individuals and 84% of the patients responded to at least one antigen.  Antibody responses against specific TAA in Stage I‐III correlated with poor prognosis / shorter progression free survival. |
| (Karagiannis et al., 2015) | Melanoma Patients  167 patients:  35 stage I  44 stage II  48 stage III  40 stage IV  Controls  104 healthy | Luminex bead array assay kits for assessments of IgG4 subclass antibodies | IgG_4_ subtype | IgG_4_ predicted risk of overall disease progression (combined Stages I–IV) as well as progression in local disease (Stages I–II). |
| (Zaenker et al., 2018) | Melanoma Patients  124 early stage  Controls  121 healthy | Immunome™ Protein Array | 1627 proteins on the array | A combination of 10 autoantibody biomarkers (ZBTB7B, PRKCH, TP53, PCTK1, PQBP1, UBE2V1, IRF4, MAPK8_tv2, MSN and TPM1) that, as a panel, displays a sensitivity of 79%, specificity of 84% and an AUC of 0.828 for primary melanoma detection. |
| (Fassler et al., 2019) | Melanoma patients  Cohort 1 – 20 patients with stage IV melanoma  Cohort 2 – 21 patients with stage IV melanoma  Controls  8 patients with NSCLC | ELISA detecting IgG | MelanA, NY-ESO-1, TRP1, TRP2, GP100 | Higher pre-treatment antigen-specific antibody levels in responders to ICI compared to non-responders.  Significantly Longer OS in patients with elevated levels of anti-NY-ESO-1 antibodies and anti-MelanA antibodies in both cohorts. |
| (Diem et al., 2019) | Melanoma patients  49 patients with metastatic melanoma | Behring nephelometer II (BNII) | Levels of total IgG and individual IgG subclasses | IgG2 levels were significantly higher in pre-treatment serum from responders vs. non-responders to ICI. No difference in any other subclasses or total IgG. |
| (Bartels et al., 2019) | Melanoma patients  157 melanoma patients (24 had advanced/metastatic melanoma) | Indirect immunofluorescence | IgA/IgM/IgG autoantibodies against neuronal antigens | Neuronal autoantibodies were found in 22.3% of patients.  Patients that harboured neuronal autoantibodies had significantly impaired overall cognitive performance. |
| (Ene et al., 2020) | Melanoma patients  160 primary melanoma patients  170 metastatic melanoma  Controls  50 healthy controls. | immunoblot technique (EUROLine kit) | IgG and IgM against the ganglioside antigens GM1, -GM2, -GM3, -GD1a, -GD1b, -GT1b, -GQ1b | The levels of antiganglioside abs in patients with metastatic melanoma was significantly lower than that of primary melanoma, while they were absent in healthy controls. |
| (Kessler et al., 2019) | Prospective study with 29,876 individuals without melanoma with mean follow-up time of 15.3 years during which 162 participants developed melanoma | HITACHI 911 automatic analyser | Total serum levels of IgG, IgA and IgM | Pre-diagnostic serum IgG levels was inversely associated with risk of developing melanoma, although not statistically significant. No associations were found with serum IgA or IgM. |

**References**

BARTELS, F., STRÖNISCH, T., FARMER, K., RENTZSCH, K., KIECKER, F. & FINKE, C. 2019. Neuronal autoantibodies associated with cognitive impairment in melanoma patients. *Ann Oncol,* 30**,** 823-9.

BAZHIN, A. V., WIEDEMANN, N., SCHNÖLZER, M., SCHADENDORF, D. & EICHMÜLLER, S. B. 2007. Expression of GAGE family proteins in malignant melanoma. *Cancer Letters,* 251**,** 258-267.

DIEM, S., FASSLER, M., BOMZE, D., ALI, O. H., BERNER, F., NIEDERER, R., HILLMANN, D., MANGANA, J., LEVESQUE, M. P., DUMMER, R., RISCH, L., RECHER, M., RISCH, M. & FLATZ, L. 2019. Immunoglobulin G and Subclasses as Potential Biomarkers in Metastatic Melanoma Patients Starting Checkpoint Inhibitor Treatment. *J Immunother,* 42**,** 89-93.

ENE, C. D., TAMPA, M., NICOLAE, I., MITRAN, C. I., MITRAN, M. I., MATEI, C., CARUNTU, A., CARUNTU, C. & GEORGESCU, S. R. 2020. Antiganglioside Antibodies and Inflammatory Response in Cutaneous Melanoma. *J Immunol Res,* 2020**,** 2491265.

FASSLER, M., DIEM, S., MANGANA, J., HASAN ALI, O., BERNER, F., BOMZE, D., RING, S., NIEDERER, R., DEL CARMEN GIL CRUZ, C., PEREZ SHIBAYAMA, C. I., KROLIK, M., SIANO, M., JOERGER, M., RECHER, M., RISCH, L., GUSEWELL, S., RISCH, M., SPEISER, D. E., LUDEWIG, B., LEVESQUE, M. P., DUMMER, R. & FLATZ, L. 2019. Antibodies as biomarker candidates for response and survival to checkpoint inhibitors in melanoma patients. *J Immunother Cancer,* 7**,** 50.

GOGAS, H., IOANNOVICH, J., DAFNI, U., STAVROPOULOU-GIOKAS, C., FRANGIA, K., TSOUTSOS, D., PANAGIOTOU, P., POLYZOS, A., PAPADOPOULOS, O., STRATIGOS, A., MARKOPOULOS, C., BAFALOUKOS, D., PECTASIDES, D., FOUNTZILAS, G. & KIRKWOOD, J. M. 2006. Prognostic significance of autoimmunity during treatment of melanoma with interferon. *N Engl J Med,* 354**,** 709-18.

HODI, F. S., SCHMOLLINGER, J. C., SOIFFER, R. J., SALGIA, R., LYNCH, T., RITZ, J., ALYEA, E. P., YANG, J., NEUBERG, D., MIHM, M. & DRANOFF, G. 2002. ATP6S1 elicits potent humoral responses associated with immune-mediated tumor destruction. *Proceedings of the National Academy of Sciences,* 99**,** 6919-6924.

HUANG, S. K., OKAMOTO, T., MORTON, D. L. & HOON, D. S. 1998. Antibody responses to melanoma/melanocyte autoantigens in melanoma patients. *J Invest Dermatol,* 111**,** 662-7.

KARAGIANNIS, P., VILLANOVA, F., JOSEPHS, D. H., CORREA, I., VAN HEMELRIJCK, M., HOBBS, C., SAUL, L., EGBUNIWE, I. U., TOSI, I., ILIEVA, K. M., KENT, E., CALONJE, E., HARRIES, M., FENTIMAN, I., TAYLOR-PAPADIMITRIOU, J., BURCHELL, J., SPICER, J. F., LACY, K. E., NESTLE, F. O. & KARAGIANNIS, S. N. 2015. Elevated IgG4 in patient circulation is associated with the risk of disease progression in melanoma. *Oncoimmunology,* 4**,** e1032492.

KESSLER, A., SOLLIE, S., KARAGIANNIS, S. N., WALLDIUS, G., HAMMAR, N. & VAN HEMELRIJCK, M. 2019. Serum IgG Is Associated With Risk of Melanoma in the Swedish AMORIS Study. *Front Oncol,* 9.

LITVAK, D. A., GUPTA, R. K., YEE, R., WANEK, L. A., YE, W. & MORTON, D. L. 2004. Endogenous immune response to early- and intermediate-stage melanoma is correlated with outcomes and is independent of locoregional relapse and standard prognostic factors. *J Am Coll Surg,* 198**,** 27-35.

LIU, Y., HE, J., XIE, X., SU, G., TEITZ-TENNENBAUM, S., SABEL, M. S. & LUBMAN, D. M. 2010. Serum autoantibody profiling using a natural glycoprotein microarray for the prognosis of early melanoma. *J Proteome Res,* 9**,** 6044-51.

MAIRE, C., VERCAMBRE-DARRAS, S., DEVOS, P., D’HERBOMEZ, M., DUBUCQUOI, S. & MORTIER, L. 2013. Metastatic melanoma: spontaneous occurrence of auto antibodies is a good prognosis factor in a prospective cohort. *Journal of the European Academy of Dermatology and Venereology,* 27**,** 92-96.

NESTEROVA, M., JOHNSON, N., CHEADLE, C. & CHO-CHUNG, Y. S. 2006. Autoantibody biomarker opens a new gateway for cancer diagnosis. *Biochimica et Biophysica Acta (BBA) - Molecular Basis of Disease,* 1762**,** 398-403.

SABEL, M. S., LIU, Y., GRIFFITH, K. A., HE, J., XIE, X. & LUBMAN, D. M. 2011. Clinical utility of serum autoantibodies detected by protein microarray in melanoma. *Int J Proteomics,* 2011**,** 413742.

SHIMBO, T., TANEMURA, A., YAMAZAKI, T., TAMAI, K., KATAYAMA, I. & KANEDA, Y. 2010. Serum Anti-BPAG1 Auto-Antibody Is a Novel Marker for Human Melanoma. *PLoS ONE,* 5**,** e10566.

STOCKERT, E., JÄGER, E., CHEN, Y.-T., SCANLAN, M. J., GOUT, I., KARBACH, J., ARAND, M., KNUTH, A. & OLD, L. J. 1998. A Survey of the Humoral Immune Response of Cancer Patients to a Panel of Human Tumor Antigens. *Journal of Experimental Medicine,* 187**,** 1349-1354.

VERCAMBRE-DARRAS, S., DUBUCQUOI, S., FAJARDY, I., PIETTE, F. & MORTIER, L. 2007. Does spontaneous autoimmunity improve survival in visceral metastatic melanoma? *British Journal of Dermatology,* 157**,** 413-415.

YUAN, J., ADAMOW, M., GINSBERG, B. A., RASALAN, T. S., RITTER, E., GALLARDO, H. F., XU, Y., POGORILER, E., TERZULLI, S. L., KUK, D., PANAGEAS, K. S., RITTER, G., SZNOL, M., HALABAN, R., JUNGBLUTH, A. A., ALLISON, J. P., OLD, L. J., WOLCHOK, J. D. & GNJATIC, S. 2011. Integrated NY-ESO-1 antibody and CD8+ T-cell responses correlate with clinical benefit in advanced melanoma patients treated with ipilimumab. *Proceedings of the National Academy of Sciences,* 108**,** 16723-16728.

ZAENKER, P., LO, J., PEARCE, R., CANTWELL, P., COWELL, L., LEE, M., QUIRK, C., LAW, H., GRAY, E. & ZIMAN, M. 2018. A diagnostic autoantibody signature for primary cutaneous melanoma. *Oncotarget,* 9**,** 30539-30551.

ZIPPELIUS, A., GATI, A., BARTNICK, T., WALTON, S., ODERMATT, B., JAEGER, E., DUMMER, R., UROSEVIC, M., FILONENKO, V., OSANAI, K., MOCH, H., CHEN, Y. T., OLD, L. J., KNUTH, A. & JAEGER, D. 2007. Melanocyte differentiation antigen RAB38/NY-MEL-1 induces frequent antibody responses exclusively in melanoma patients. *Cancer Immunol Immunother,* 56**,** 249-58.

ZORNIG, I., HALAMA, N., LORENZO BERMEJO, J., ZIEGELMEIER, C., DICKES, E., MIGDOLL, A., KAISER, I., WATERBOER, T., PAWLITA, M., GRABE, N., UGUREL, S., SCHADENDORF, D., FALK, C., EICHMULLER, S. B. & JAGER, D. 2015. Prognostic significance of spontaneous antibody responses against tumor-associated antigens in malignant melanoma patients. *Int J Cancer,* 136**,** 138-51.
