# Supplementary material for: A B‐cell or a key player? The different roles of B‐cells and antibodies in melanoma
Source: Pigment Cell Melanoma Res. 2022 Mar 4;35(3):303–19. doi: 10.1111/pcmr.13031 (PMC9314792; doi:10.1111/pcmr.13031)
Supplement: Supplementary file 4 — Supplementary Material [file PCMR-35-303-s002.docx]

## Foreword

This Supplementary material is provided to give an overview of one of the important features of the B-cells: the ability to produce and refine antibodies. This is intended as a general description to facilitate the understanding of the B-cell mediated antibody response in melanoma.

## Immunoglobulins produced by B-cells

B-cells produce antibodies (immunoglobulins; Ig), which are glycoproteins individually capable of binding to specific peptide sequences. The first antibodies made by a newly formed B-cell are inserted into the plasma membrane (~1x10^5^/cell) and serve as the B-cell receptor (BCR) for antigen. Upon B-cell activation, Ig heavy-chain RNA transcription is altered to contain a hydrophilic C-terminus, enabling secretion; i.e. antibodies are the secreted forms of BCRs. As B-cells mature, the Ig-class they produce is altered, providing different functionalities in response to pathogen. There are five antibody isotypes (IgM, IgD, IgA, IgE and IgG; Supplementary Figure 1), which vary in structure, body distribution, the cells they interact with and their cognate receptors. Of relevance to this review, there are four IgG subclasses (IgG_1 - 4_) determined by differences in the Fc domain and the number of disulfide bonds in the hinge region (Schauer et al., 2003); each subclass varies in the effector functions they initiate (reviewed in Vidarsson et al., 2014).

## Class switch recombination

Activated B-cells irreversibly change their immunoglobulin isotype from IgM or IgD to IgG, IgA or IgE by ‘class switch recombination’ (CSR) (Figure 1). CSR involves intrachromosomal rearrangement of the constant region genes of the Ig heavy chain, which determines the antibody isotype. Somatic hypermutation (SHM) results in the selection of B-cells expressing Ig with an enhanced ability to recognise and bind a specific antigen. CSR and SHM both rely on the enzyme ‘activation-induced cytidine deaminase’ (AID). CSR occurs as early as the initial interaction with cognate T-cell help (T_H_) at the border between the T-cell and B-cell zones in secondary and tertiary lymphoid structures (SLS and TLS, respectively (Roco et al., 2019)), which are described in greater detail in the main review.

## Mechanisms of antibody effects

The function of antibodies produced are hypothesised based on known responses to pathogens, but are yet to be demonstrated in response to melanoma. These include alteration of function of targets on cells, opsonisation, activation of the complement cascade, activation of antibody-dependent cell-mediated phagocytosis (ADCP), or activation of antibody-dependent cell-mediated cytotoxicity (ADCC). ADCC is triggered when sufficient antigen has been bound for the Fc domain of immunoglobulin to bind and cross-link Fc receptors (FcRs) on the surface of effector cells (e.g. NK cells, neutrophils and eosinophils); Supplementary Figure 1. The FcR are classified based on the type of antibody that they recognise; e.g. the IgG-binding FcγR consists of four different subtypes, FcγRI, FcγRIIa, FcγRIIb and FcγRIII, each with varying affinity for IgG subclasses and differing expression patterns on leukocytes. FcγRs can be activating (FcγRI, FcγRIIa and FcγRIII) or inhibitory (FcγRIIb).

**References**

ROCO, J. A., MESIN, L., BINDER, S. C., NEFZGER, C., GONZALEZ-FIGUEROA, P., CANETE, P. F., ELLYARD, J., SHEN, Q., ROBERT, P. A., CAPPELLO, J., VOHRA, H., ZHANG, Y., NOWOSAD, C. R., SCHIEPERS, A., CORCORAN, L. M., TOELLNER, K. M., POLO, J. M., MEYER-HERMANN, M., VICTORA, G. D. & VINUESA, C. G. 2019. Class-Switch Recombination Occurs Infrequently in Germinal Centers. *Immunity,* 51**,** 337-350.e7.

SCHAUER, U., STEMBERG, F., RIEGER, C. H., BORTE, M., SCHUBERT, S., RIEDEL, F., HERZ, U., RENZ, H., WICK, M., CARR-SMITH, H. D., BRADWELL, A. R. & HERZOG, W. 2003. IgG subclass concentrations in certified reference material 470 and reference values for children and adults determined with the binding site reagents. *Clin Chem,* 49**,** 1924-9.

VIDARSSON, G., DEKKERS, G. & RISPENS, T. 2014. IgG subclasses and allotypes: from structure to effector functions. *Front Immunol,* 5**,** 520.
